# Rapid cyclic stretching of cultured human visceral smooth muscle cells promotes a synthetic, proinflammatory phenotype

**DOI:** 10.1172/jci.insight.188669

**Published:** 2025-09-16

**Authors:** Sharon M. Wolfson, Katherine Beigel, Sierra E. Anderson, Brooke Deal, Molly Weiner, Se-Hwan Lee, Deanne M. Taylor, Su Chin Heo, Robert O. Heuckeroth, Sohaib K. Hashmi

**Affiliations:** 1 The Children’s Hospital of Philadelphia Research Institute and the Abramson Research Center, Philadelphia, Pennsylvania, USA.; 2 Department of Pediatrics, Perelman School of Medicine, University of Pennsylvania, Philadelphia, Pennsylvania, USA.; 3 The Department of Biomedical and Health Informatics, The Children’s Hospital of Philadelphia, Philadelphia, Pennsylvania, USA.; 4 Perelman School of Medicine at the University of Pennsylvania, Philadelphia, Pennsylvania, USA.; 5 McKay Orthopaedic Research Laboratory, Department of Orthopaedic Surgery, Perelman School of Medicine, University of Pennsylvania, Philadelphia, Pennsylvania, USA.; 6 Department of Bioengineering, The University of Pennsylvania School of Engineering and Applied Science, Philadelphia, Pennsylvania, USA.; 7 Center for Engineering Mechanobiology, University of Pennsylvania, Philadelphia, Pennsylvania, USA.; 8 Translational Musculoskeletal Research Center, Corporal Michael J. Crescenz VA Medical Center, Philadelphia, Pennsylvania, USA.; 9 Department of Internal Medicine, Division of Gastroenterology and Hepatology, Hospital of the University of Pennsylvania, Philadelphia, Pennsylvania, USA.

**Keywords:** Cell biology, Gastroenterology, Muscle biology, Cytokines, Fibrosis, Muscle

## Abstract

Bowel smooth muscle experiences mechanical stress constantly during normal function and pathologic mechanical stressors in disease states. We tested the hypothesis that pathologic mechanical stress could alter transcription to induce smooth muscle phenotypic class switching. To test this hypothesis, primary human intestinal smooth muscle cells (HISMCs), seeded on electrospun aligned poly-ε-caprolactone nano-fibrous scaffolds, were subjected to pathologic, high-frequency (1 Hz) uniaxial 3% cyclic stretch (loaded) or kept unloaded in culture for 6 hours. RNA-Seq, quantitative PCR (qPCR), and quantitative IHC defined loading-induced changes in gene expression. NicheNet predicted how differentially expressed genes might affect HISMCs and other bowel cells. These studies show loading induced differential expression of 4,537 HISMC genes. Loaded HISMCs had a less contractile phenotype, with increased expression of synthetic SMC genes, proinflammatory cytokines, and altered expression of axon guidance molecules, growth factors, and morphogens. Many differentially expressed genes encode secreted ligands that could act cell autonomously on smooth muscle and on other cells in the bowel wall. These data show that HISMCs undergo remarkably rapid phenotypic plasticity in response to mechanical stress that may convert contractile HISMCs into proliferative fibroblast-like cells or proinflammatory cells. These mechanical stress–induced changes in HISMC gene expression may be relevant for human bowel disease.

## Abstract 2

Cyclically stretching bowel smooth muscle cells at a much higher frequency than what the bowel normally experiences induces a proinflammatory state in these cells.

## Graphical abstract

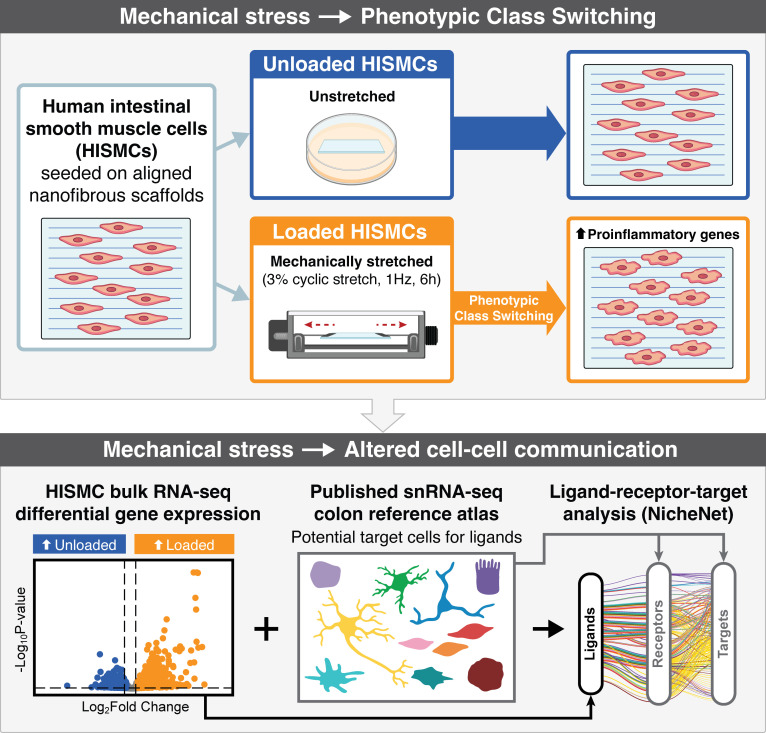

## Introduction

The gastrointestinal (GI) tract is constantly moving to digest and absorb nutrients and to eliminate waste. This movement creates mechanical stress that can be sensed by cells and may alter gene expression through mechanotransduction pathways (1–4). In the bowel, pathologic radial or longitudinal force occurs from mechanical obstruction (stricture, web, volvulus, adhesions), motility disorders (achalasia, gastroparesis, Hirschsprung disease, chronic intestinal pseudo-obstruction [CIPO]), and surgical manipulation. Similar to physiologic mechanical forces, pathological mechanical forces may also induce transcriptional changes that alter cell phenotypes (1, 4, 5). While many bowel cell types respond to mechanical cues (4), we hypothesized that unusual mechanical stressors might particularly affect gene expression in bowel (visceral) smooth muscle cells (SMCs), altering cell fate. These changes in visceral SMC fate were predicted based on decreased contractile smooth muscle marker expression in pediatric CIPO bowel (6) and by extrapolating from vascular smooth muscle, which undergo “phenotypic class switching” to synthetic, proliferative phenotypes in response to injury (6–8). This phenotypic class switching for vascular SMCs is thought to be protective but is also an important pathophysiologic mechanism in hypertension, ischemic vascular disease, and atherosclerosis.

Phenotypic class switching is not well studied in visceral SMCs, and differences between visceral and vascular SMCs may make extrapolation inappropriate. However, prior studies suggest visceral SMCs also change fate in response to specific mechanical stressors. For example, partial intestinal obstruction increases SMC expression of COX-2 (PTGS2), mPGES-1, and PGE2 (9) in vivo, while stretch in vitro of primary colon SMCs increases IL-8, IL-6, MCP1, iNOS, COX2, BDNF, and NGF (10). Mechanical stress also induces human fetal visceral SMC expression of profibrotic mediators, including TGF-β1 and α1 collagen (11), markers of synthetic SMCs.

To test the hypothesis that mechanical stress could rapidly alter gene expression in visceral SMCs, and to gain insight into early changes in SMC phenotype in response to mechanical stress, we evaluated gene expression in cultured human intestinal SMCs (HISMCs) after 6 hours in culture with or without cyclic stretching. We used low amplitude, high-frequency mechanical stress (3%, 1 Hz), a frequency up to 10-fold greater than physiologic bowel contraction. This pathologic stress rapidly altered expression of 4,537 genes (adjusted *P* < 0.05 for loaded [stretched] versus unloaded cells). Of these genes, 2,500 had log_2_ fold change > 0.48 or < –0.48. Compared with unloaded HISMCs, loaded cells had increased expression of synthetic phenotype SMCs genes, increased production of many cytokines, chemokines, cytokine receptors, axon guidance molecules, junctional proteins, and altered levels of signaling molecules predicted to act on nearby bowel cells. Collectively, these data suggest that bowel SMC phenotype, in part, depends on unique physical forces experienced as nutrients move through bowel and waste is eliminated, in response to injury or disease, or as the body changes position (e.g., bending, running, breathing). This suggests that even brief pathologic mechanical insult may profoundly affect visceral smooth muscle phenotype. Furthermore, NicheNet analyses suggest that mechanotransduction-induced phenotypic changes in smooth muscle gene expression may increase secretion of ligands that affect many nearby bowel cells on a time scale much longer than the original insult.

## Results

### HISMCs grown on aligned scaffolds have more smooth muscle myosin heavy chain 11 (MYH11) protein and less VIM mRNA than HISMCs grown on nonaligned scaffolds.

**Figure 1:**
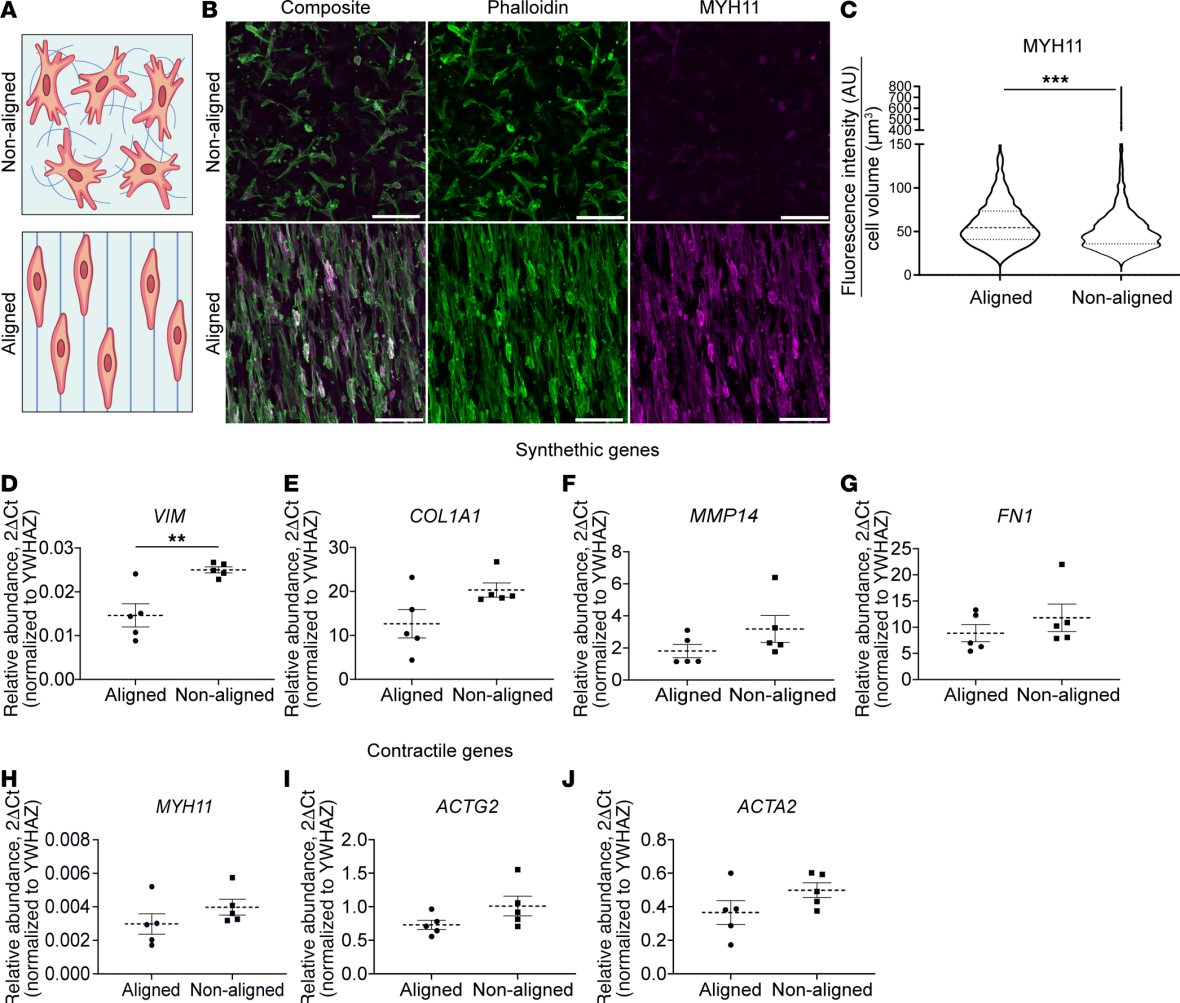
HISMC cultured on aligned nanofibrous spun scaffolds had more MYH11 protein and less *VIM* mRNA compared with HISMC cultured on nonaligned scaffolds. (**A**) Schematic of nonaligned (top) and aligned (bottom) PCL scaffolds. (**B**) Confocal Z-stack maximum intensity projections of HISMCs stained with antibodies to smooth muscle myosin (MYH11, magenta) and F-actin (Phalloidin-Alexa Fluor 488, green) after culture on nonaligned (top) or aligned (bottom) scaffolds for 72 hours. Scale bar: 100 μm. (**C)** MYH11 antibody staining was brighter in HISMCs cultured 72 hours on aligned scaffolds compared with HISMC cultured on nonaligned scaffolds (median [interquartile range] aligned: 54.33 AU [32.6 AU], nonaligned: 47.6 AU [28.28 AU], Mann-Whitney test, *P* < 0.0001, *n* = 3). (**D**–**J**) qPCR analyses for mRNA levels of smooth muscle synthetic genes (*VIM*, *COL1A1*, *MMP14*, *FN1*) showed increased VIM expression in HISMCs grown on nonaligned scaffolds (**D**), with similar expression of other synthetic genes. qPCR analyses demonstrated mRNA levels of smooth muscle contractile genes (*MYH11*, *ACTG2*, *ACTA2*) were similar for HISMCs grown on nonaligned and aligned scaffolds. *VIM* (mean ± SEM aligned: 0.01462 ± 0.002633, mean nonaligned: 0.025 ± 0.0002020, *P* = 0.0052, *n* = 5). *ACTA2* (mean ± SEM aligned: 0.3656 ± 0.07053, mean nonaligned: 0.4986 ± 0.04450, *P* = 0.1494, *n* = 5). *MYH11* (mean ± SEM aligned: 0.002982 ± 0.0006099, mean nonaligned: 0.003978 ± 0.0004698, *P* = 0.2322, *n* = 5). *COL1A1* (median [interquartile range], aligned: 10.38 [12.679], nonaligned: 18.95 [4.61], *P* = 0.0952, *n* = 5). *FN1* (median [interquartile range], aligned: 6.985 [6.93], nonaligned: 10.21 [8.483], *P* = 0.4206, *n* = 5). *ACTG2* (mean ± SEM aligned: 0.7499 ± 0.06910, mean nonaligned: 1.009 ± 0.1477, *P* = 0.1249, *n* = 5). *MMP14* (median [interquartile range], aligned: 1.16 [1.624], nonaligned: 2.319 [2.898], *P* = 0.2222, *n* = 5). **P < 0.01, ***P < 0.001.

Our initial goal was to test the hypothesis that pathological mechanical stress acutely alters gene expression in contractile bowel SMC. One challenge is that SMCs cultured on hard plastic rapidly undergo phenotypic class switching from a “contractile” (MYH11-expressing) to a “synthetic” phenotype that produces extracellular matrix (ECM), migrates, and proliferates (7, 12). To study the effects of mechanical stress in a more contractile phenotype cell, we seeded HISMCs onto electrospun poly-caprolactone (PCL) scaffolds (13) coated with laminin, an ECM protein that promotes the contractile SMC phenotype (14, 15) (Figure 1, A and B). One set of PCL scaffolds was spun to have aligned fibers to promote growth of elongated spindle-shaped SMCs reported to be more contractile (16, 17). In parallel, HISMCs were cultured on laminin-coated PCL scaffolds with nonaligned fibers (Figure 1, A and B). After 72 hours with scaffolds floating freely in HISMC media, cells were fixed and stained with antibodies to MYH11, a contractile apparatus protein prominently produced in mature contractile phenotype SMCs. Pixel intensity measurements showed HISMCs grown on aligned scaffolds averaged (mean) 14% more MYH11 protein than HISMC grown on nonaligned scaffolds (aligned: 54.33 arbitrary units [AU] [32.6 AU]; nonaligned: 47.6 AU [28.28 AU], median [interquartile range]) (Mann-Whitney *U* test, *P* < 0.0001, *n* = 3) (Figure 1, B and C). Vimentin (*VIM*) mRNA, a synthetic SMC marker (18), was also less abundant in HISMCs cultured on aligned compared with nonaligned scaffolds (*P* = 0.0052, *n* = 5) (Figure 1D). In contrast, mRNA for ECM-related (*COL1A1*, *MMP14*, *FN1*) and contractile apparatus genes (*MYH11*, *ACTG2*, *ACTA2*) were statistically equivalent in HISMCs cultured on aligned versus nonaligned scaffolds (Figure 1, E–J). Based on these findings, further experiments used aligned scaffolds.

### Dynamic loading (cyclic stretching) of HISMCs leads to marked changes in gene expression.

**Figure 2:**
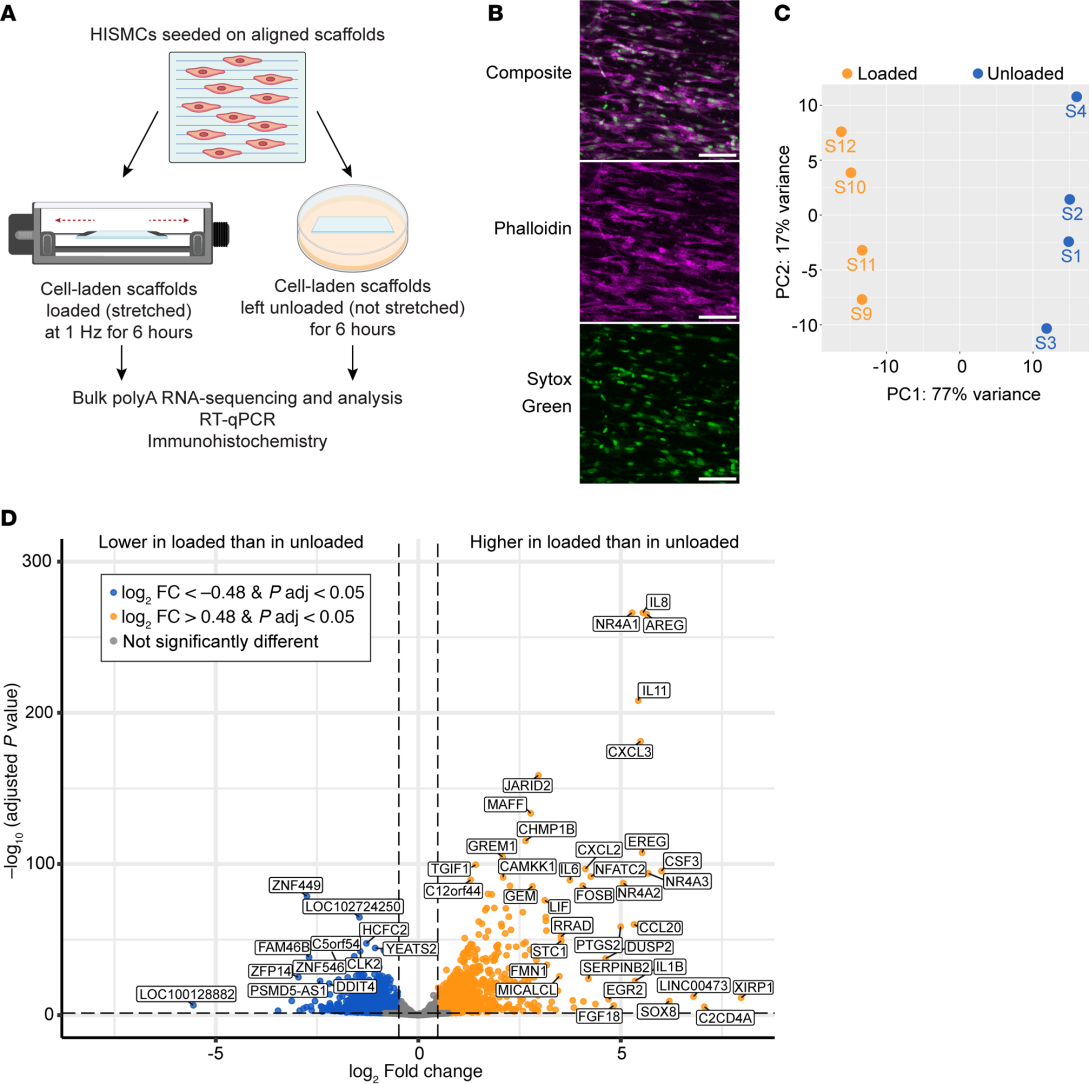
Dynamic loading for 6 hours led to many changes in gene expression. (**A**) Schematic of experimental design. (**B**) Sum-of-slices Z-projection of 20× confocal image of HISMCs on aligned scaffolds stained for F-actin (Phalloidin, magenta) with nuclei labeled with Sytox Green. Scale bar: 100 μm. (**C**) PCA plot (based on the top 500 most variable genes) showing loaded versus unloaded HISMCs differ significantly in gene expression. Orange dots, loaded; blue dots, unloaded. (**D**) Volcano plot of differentially expressed genes between loaded versus unloaded HISMCs, from DESeq2 analysis. Log_2_fold change (log_2_FC) cutoff > 0.48 or < –0.48 (vertical dotted lines). False Discovery Rate (FDR) cutoff = 0.05 (horizontal dotted line). Orange dots indicate differentially expressed genes (FDR < 0.05) with log_2_FC > 0.48 (1239 genes up in loaded), and blue dots indicate differential expressed genes (FDR < 0.05) with log_2_FC < –0.48 (1,261 genes up in unloaded).

To identify early gene expression changes in response to pathologic stretch, HISMCs cultured 72 hours on aligned PCL scaffolds were subjected to cyclic uniaxial stretch (loaded) along the long axis of the cell (3% stretch, 1 Hz, 6 hours). Unloaded control scaffolds were maintained free-floating in fresh culture media for 6 hours (Figure 2A). Scaffolds were then stained (Figure 2B) or dissolved in Trizol for RNA-Seq. Bulk RNA-Seq demonstrated clear separation of loaded (stretched) versus unloaded (free-floating) HISMCs using Principal Component Analysis (PCA) (*n* = 4 per group; Figure 2C). Differential expression analysis using DESeq2 identified 1,239 mRNA that were more abundant (log_2_FC > 0.48) and 1,261 mRNA less abundant (log_2_FC < –0.48) in loaded compared with unloaded HISMCs (adjusted *P* < 0.05, DESeq2). This gene set includes markers of contractile or synthetic SMC phenotypes, inflammatory mediators, TGF-β superfamily genes, axon guidance molecules, cytoskeletal proteins, cell-cell junctional proteins, and cell-ECM interacting proteins (Figure 2D). In addition, gene set enrichment analysis (GSEA) using Hallmark gene sets from the Human Molecular Signatures Database (MSigDB) highlighted several cytokine and inflammation pathways with normalized enrichment scores (NES) greater than 2 (Supplemental Figure 1; supplemental material available online with this article; https://doi.org/10.1172/jci.insight.188669DS1), suggesting cyclic mechanical stress induces gene expression changes associated with proinflammatory states. These pathways included “TNF alpha signaling via NFκB,” “Inflammatory Response,” “Allograft Rejection,” “IL6-JAK-STAT3 Signaling,” “IL2-STAT5 Signaling,” and “Interferon Gamma Response.”

**Figure 3:**
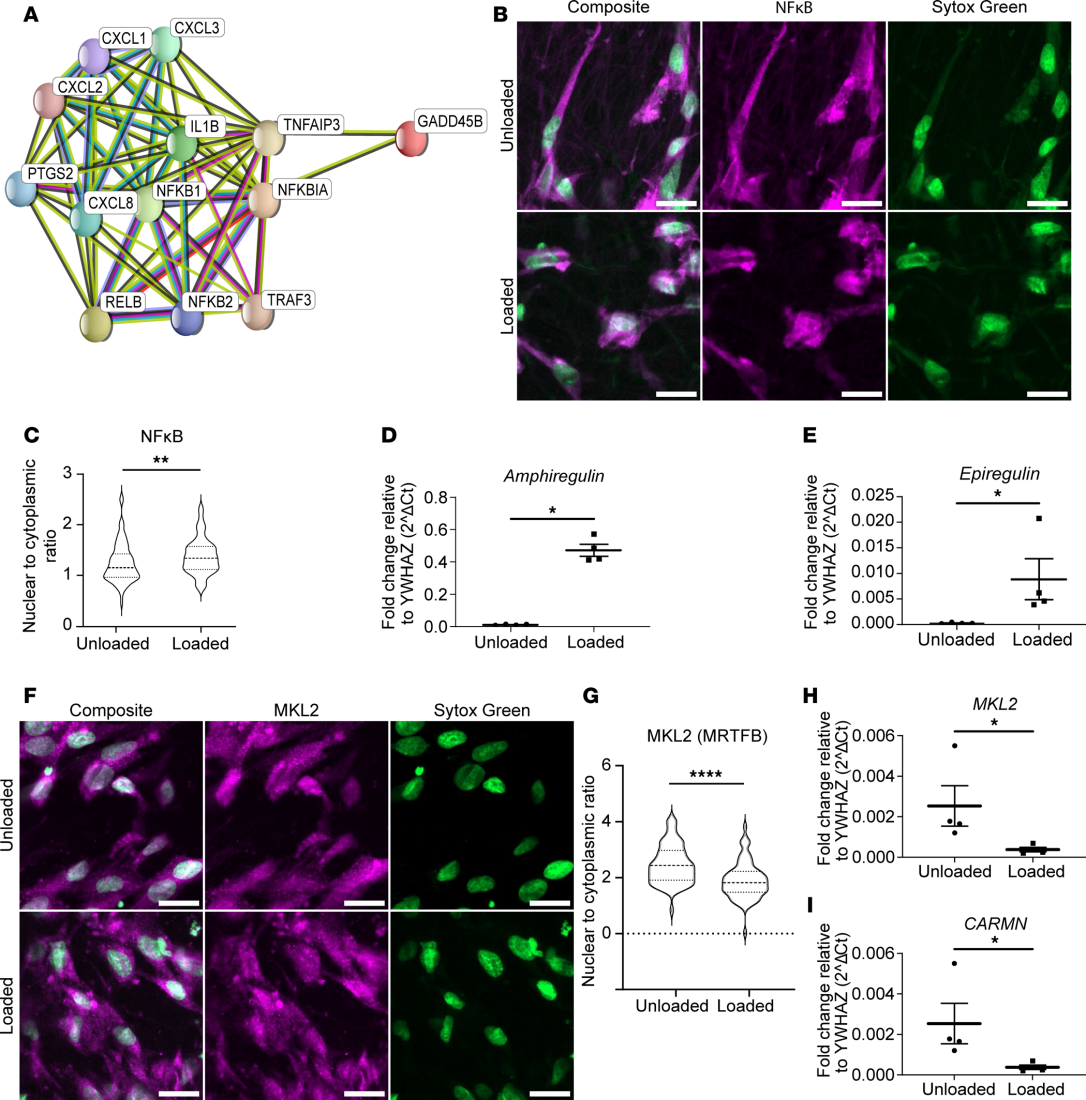
Loaded HISMCs have increased synthetic gene expression and activation of NF-κB signaling. (**A**) STRING diagram of KEGG NF-κB pathway analysis. Input included mRNA more abundant in loaded HISMCs with log_2_ fold change (log_2_FC) > 0.48 based on bulk RNA-Seq. (**B**) Representative sum-of-slices Z-projections of confocal images (63× oil objective) showing NF-κB antibody staining (magenta) and Sytox green nuclear staining. Top: Unloaded HISMCs grown on aligned scaffold. Bottom: Loaded HISMCs grown on aligned scaffold. Scale bar: 20 μm. (**C**) Quantitative analysis of antibody staining demonstrated increased NF-κB nuclear to cytoplasmic ratio in loaded compared with unloaded HISMCs (median [interquartile range] unloaded 1.152 [0.4651], loaded 1.336 [0.8271] (*P* = 0.0081, Mann Whitney), *n* = 79 cells for both groups. (**D** and **E**) qPCR analyses for mRNA levels of smooth muscle synthetic genes (*Amphiregulin*, *Epiregulin*) showed increased *Amphiregulin* and *Epiregulin* expression in loaded HISMCs. *Amphiregulin* (median [interquartile range], unloaded: 0.01114 [0.006101], loaded: 0.4518 [0.1364], *P* = 0.0286, *n* = 8). *Epiregulin* (median [interquartile range], unloaded: 0.0002173 [0.000219], loaded: 0.005386 [0.01303], *P* = 0.0286, *n* = 8). (**F**) Representative sum-of-slices Z-projections of confocal images (63× oil objective) of MKL2 stained HISMCs (red) and Sytox green nuclear staining. Top: Unloaded HISMCs grown on aligned scaffold. Bottom: Loaded HISMCs grown on aligned scaffold. Scale bar: 20 µm. (**G**) Quantitative analysis of antibody staining demonstrated an increased MKL2 nuclear to cytoplasmic ratio in unloaded compared with loaded HISMCs (median [interquartile range] unloaded 2.435 [1.066], loaded 1.820 [0.731], *P* < 0.0001, Mann Whitney, *n* = 65 cells for unloaded and *n* = 70 cells for loaded HISMCs). (**H** and **I**) qPCR analyses for mRNA of smooth muscle contractile genes showed decreased *CARMN* and *MKL2* expression in loaded HISMCs. *CARMN* (median [interquartile range], unloaded: 0.001717 [0.003256], loaded: 0.0003141 [0.0003979], *P* = 0.0286, *n* = 8). *MKL2* (median [interquartile range], unloaded: 0.001717 [0.003256], loaded: 0.0003141 [0.0003979], *P* = 0.0286, *n* = 8). *P < 0.05, **P < 0.01.

Consistent with the hypothesis that loading induced a proinflammatory state, STRING classification using KEGG pathways to characterize the 500 most differentially regulated genes (based on adjusted *P* values) identified 13 NF-κB pathway genes more abundant in loaded than in unloaded HISMCs (Figure 3A). To determine if this reflected increased NF-κB signaling, we used IHC and discovered more nuclear NF-κB in loaded compared with unloaded HISMCs (Figure 3, B and C). Since NF-κB (NFKB1, NFKB2) also promotes a synthetic phenotype in SMCs by repressing myocardin, the master regulator for SMC contractile phenotype (19), we hypothesized that loaded HISMCs might have a more synthetic phenotype than unloaded HISMCs. Consistent with this hypothesis, loaded HISMCs had higher levels of mRNA for many synthetic SMC phenotype genes compared with unloaded HISMCs, including *EREG* (20), *AREG* (21), *KLF4* (22), *PDGFA* (23), *EPHA2* (24), *ETS1*, *ETS2*, *ELF1* (25), *POU2F2* (26), and *THBS1* (27) (Table 1 and Figure 3, D and E). Loaded HISMCs also had less nuclear MKL2 protein compared with unloaded HISMCs (Figures 3, F and G) and less mRNA encoding MKL2 (log_2_FC = –0.5, adjusted *P* = 0.0029) (Figure 3H). MKL2 is a myocardin transcription factor family gene (also called Myocardin Related Transcription Factor B [MRTFB]) that promotes the SMC contractile phenotype (28). Furthermore, loaded HISMCs had less mRNA for *CARMN* (reported as *MIR143HG* in Supplemental Table 1) (log_2_FC = –2.06, adjusted *P* = 9.68 × 10^–5^), a long noncoding RNA critical for maintaining visceral SMC contractile function (29) (Figure 3I). Collectively, these data show our cyclic stretching paradigm promotes a synthetic, proinflammatory state in HISMCs, instead of a contractile phenotype.

**Figure 4:**
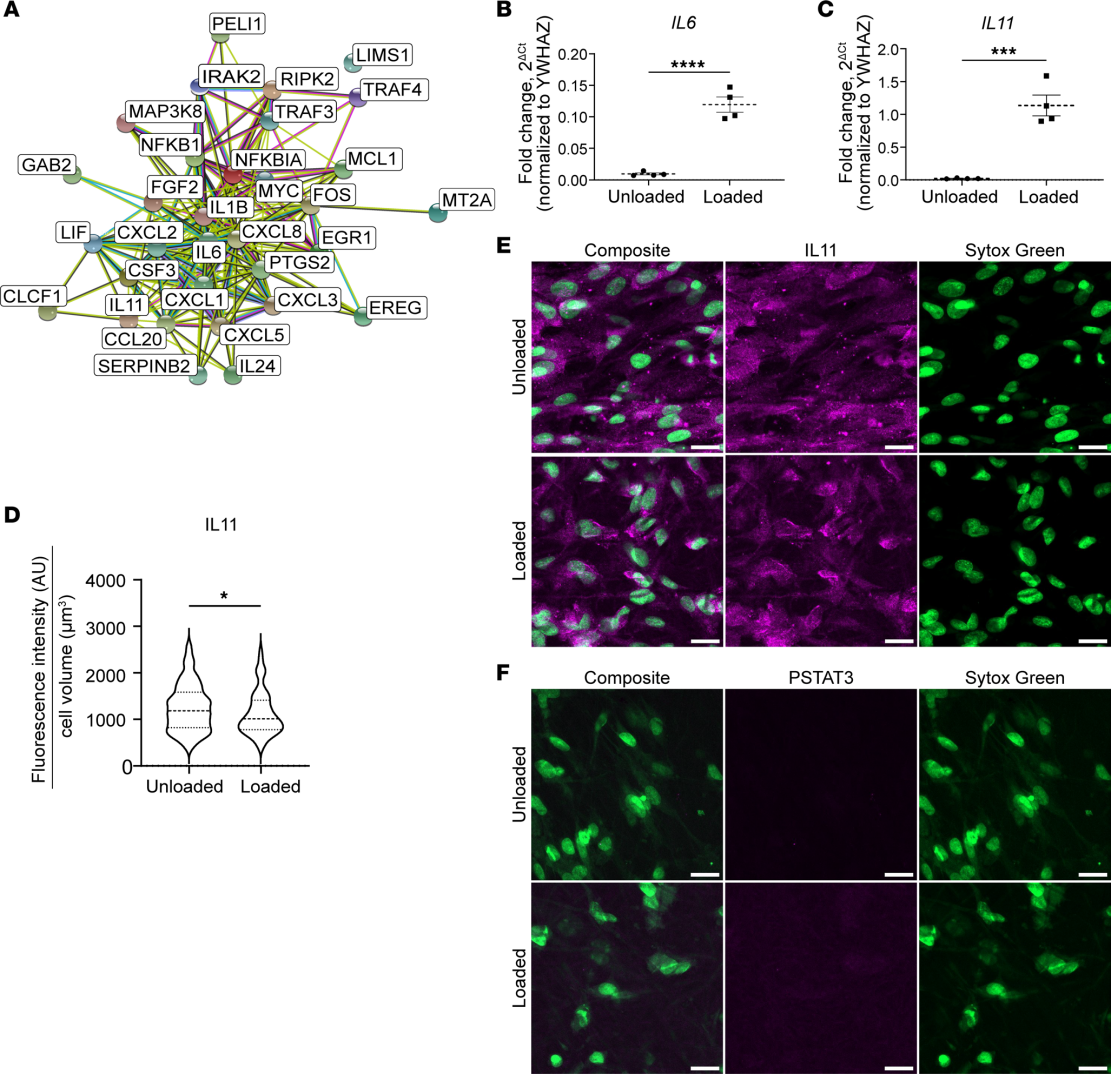
Supraphysiologic cyclical stretching stimulates production of cytokines, cytokine receptors, and chemokines. (**A**) STRING diagram of 32 genes (from top 500 differentially expressed genes (*P*_adj_ < 1 × 10^–6^, absolute value log_2_FC > 0.48 out of 1,113 genes meeting this criteria) from bulk RNA-Seq annotated as genes in GO Biological Process cytokine-mediated signaling pathway (GO:0019221) in STRING analysis. (**B**) qPCR shows 12-fold more *IL-6* mRNA in loaded compared with unloaded HISMCs (mean ± SEM unloaded: 0.009601 ± 0,001695, loaded: 0.1195 ± 0.01191, *P* < 0.0001, *n* = 4). (**C**) qPCR shows 55-fold more *IL11* mRNA in loaded compared with unloaded HISMCs (mean ± SEM unloaded: 0.02060 ± 0.002797, loaded: 1.136 ± 0.1585, *P* = 0.0004, *n* = 4). (**D**) Quantification of IL-11 IHC shows a small, statistically significant increase in pixel intensity in unloaded compared with loaded HISMCs (median [interquartile range], unloaded: 1,184 [762.6], *n* = 122; loaded: 1,012 [631.2], *n* = 99; *P* = 0.0469, Mann-Whitney). (**E**) Representative sum-of-slices Z-projections of confocal images of loaded or unloaded HISMCs stained with antibodies to IL11 (63× oil objective, confocal Z-stack). (**F**) Representative sum-of-slices Z-projections of confocal images of loaded or unloaded HISMCs stained with antibodies to phosphorylated STAT3 (PSTAT3) (63× oil objective, confocal Z-stack). PSTAT3 was not detected in HISMCs under either condition, but PSTAT3 was readily detectable in human monocyte THP-1 cell line (Supplemental Figure 2). *P < 0.05, ***P < 0.001, ****P < 0.0001.

These findings are reinforced by STRING classification of the top 500 genes (by adjusted *P* value) with absolute value log_2_FC > 0.48 using Gene Ontology (GO) Biological Process pathways (30, 31). In STRING analysis, 30 of these top 500 genes were involved in cytokine signaling (cytokine-mediated signaling pathway, GO:0019221) including *IL8*, *CXCL3*, *IL11*, *IL1B*, *CCL20*, *PTSG2*, *CXCL2*, *IL6*, *LIF*, *IL24*, *CXCL1*, *CXCL5*, and *CLCF1* (Figure 4A and Table 2). Many of these cytokines may impair intestinal motility (32–34). To validate RNA-Seq, we used quantitative PCR (qPCR) to analyze mRNA abundance for *IL6* (Figure 4B), a major proinflammatory cytokine (35), and *IL11* (Figure 4C), which promotes a synthetic phenotype in vascular smooth muscle (34). qPCR showed that *IL6* mRNA was 12-fold more abundant (*P* < 0.001, *n* = 4) in loaded versus unloaded HISMCs and *IL11* mRNA was 55-fold (*P* = 0.004, *n* = 4) more abundant in loaded HISMCs. This is similar to the 13.4-fold (log_2_FC = 3.74) elevation in *IL-6* and 43.1-fold (log_2_FC = 5.43) elevation in *IL11* based on RNA-Seq (Table 2). In contrast to mRNA, IL-11 IHC revealed lower protein levels in loaded than in unloaded HISMCs (Figures 4, D and E). In addition, phospho-STAT3, a key IL-6 signaling protein, was not detected in either loaded or unloaded HISMCs by antibody staining (Figure 4F) although we readily detected phospho-STAT3 in human THP-1 macrophages (Supplemental Figure 2). Collectively, these data show dramatic increases in many proinflammatory signaling molecules at the mRNA level after only 6 hours of pathologic stretching.

### TGF-β superfamily genes are differentially expressed in loaded versus unloaded HISMC.

**Figure 5:**
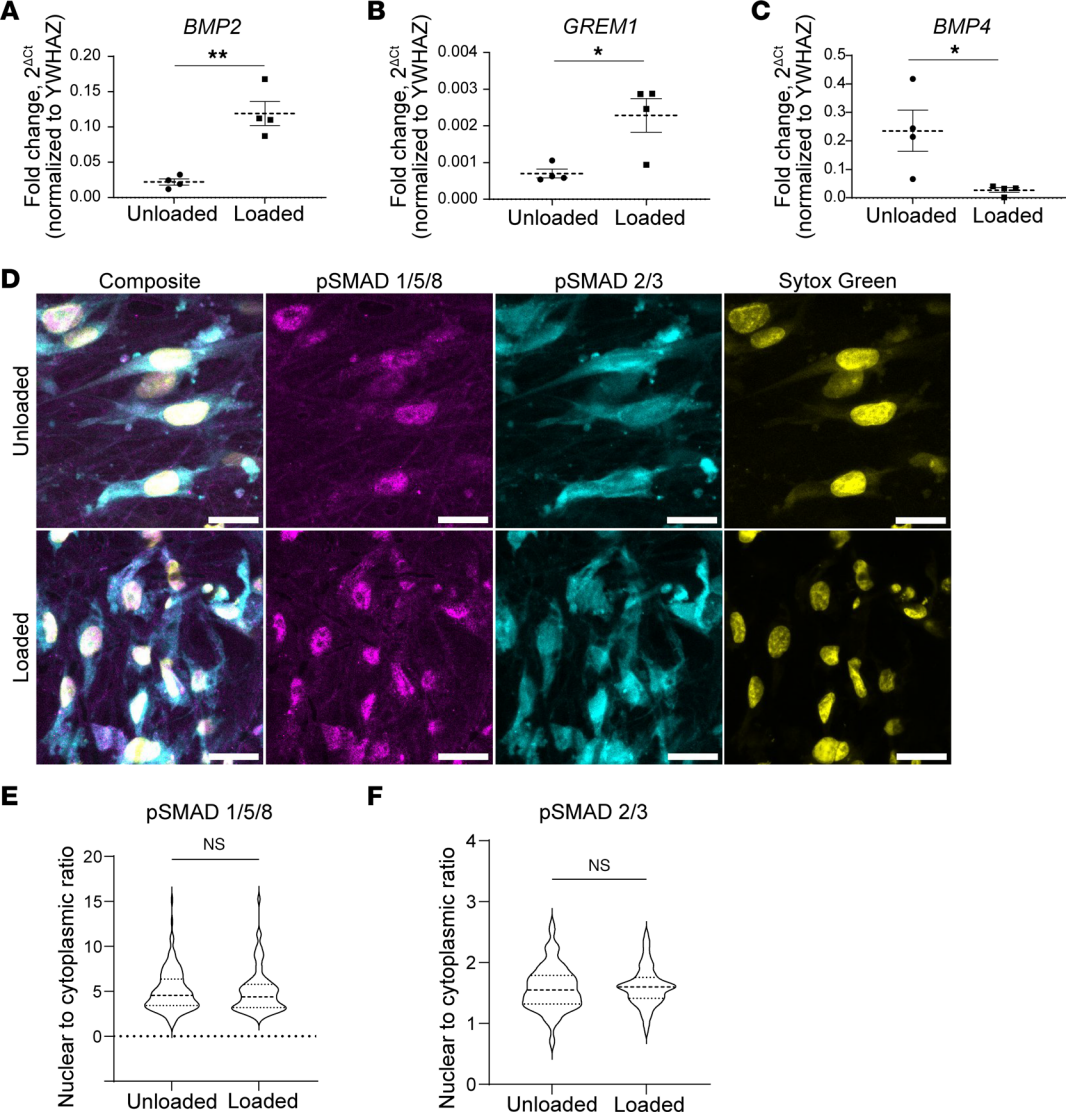
TGF-β superfamily genes are differentially expressed after HISMC loading. (**A**) qPCR confirms 5.36× increased *BMP2* in loaded HISMCs (mean ± SEM unloaded: 0.02222 ± 0.004256, loaded: 0.1191 ± 0.01708, *P* = 0.0015, *n* = 4). (**B**) qPCR showed *GREM1* mRNA is 3.16× increased in loaded HISMCs (mean ± SEM unloaded: 0.0007025 ± 0.0001186, loaded: 0.00289 ± 0.0004599, *P* = 0.0156, *n* = 4). (**C**) *BMP4* reverse transcription PCR results confirming 8.63× increased BMP4 expression in unloaded HISMCs (mean ± SEM unloaded: 0.2358 ± 0.07209, loaded: 0.02733 ± 0.008379, *P* = 0.0284, *n* = 4). (**D**) Representative images taken with confocal microscope 63× oil objective of pSMAD 1/5/8 (left) and pSMAD 2/3 (right) in unloaded and loaded HISMCs. (**E**) IHC quantification of PSMAD 1/5/8 showed no differences in nuclear to cytoplasmic staining between loaded and unloaded HISMCs (median [interquartile range], unloaded: 4.549 [2.937], *n* = 150; loaded: 4.391 [2.584], *n* = 88; *P* = 0.5339, Mann-Whitney). (**F**) IHC quantification of PSMAD 2/3 showed no differences in nuclear to cytoplasmic ratio between loaded and unloaded HISMCs (median [interquartile range], unloaded: 1.547 [0.471], *n* = 150; loaded: 1.598 [0.344], *n* = 88; *P* = 0.5099, Mann-Whitney).*P < 0.05, **P < 0.01.

TGF-β signaling has roles in smooth muscle embryogenesis and phenotypic class switching (36). Many TGF-β superfamily genes were differentially regulated by 6 hours of cyclic HISMC loading based on RNA-Seq. Loaded HISMCs had more *INHBB*, *TGFBR1*, *TGFBR3*, *SMAD7*, *TGFB1*, *BMP2*, *GREM1*, and *SMAD1* mRNA and less *TMEM100*, *SMAD6*, *BAMBI*, *BMP4*, *SMAD6*, and *BAMBI* mRNA, compared with unloaded HISMCs (Table 3). qPCR confirmed higher levels of *BMP2* (Figure 5A) and *GREM1* (Figure 5B) in loaded HISMCs and reduced *BMP4* mRNA (Figure 5C) compared with unloaded cells. Since TGF-β and BMP can alter SMC phenotype, we evaluated nuclear to cytoplasmic ratios of signaling proteins that localize to the nucleus after BMP (phospho-SMAD1/5/8) or TGF-β (phospho-SMAD2/3) receptor activation (Figure 5D). Quantitative analysis of IHC showed equivalent nuclear to cytoplasmic ratios of phospho-SMAD2/3 and phospho-SMAD1/5/8 in loaded and unloaded HISMCs (Figure 5, E and F). Collectively, these data indicate that cyclic stretching rapidly alters mRNA levels for many TGF-β superfamily genes, but the signaling pathways that these genes could activate or inhibit were not altered in HISMCs, at least at this early time point.

### Pathologic loading induces differential expression of guidance molecules and of genes needed for cell-cell and cell-ECM interactions.

Cyclic HISMC loading rapidly altered mRNA levels for many ephrins, semaphorins, netrins, and slits (Table 4). In addition to central roles in neurobiology, these axon guidance molecules play key roles in vascular SMC migration, cell proliferation, and inflammation in cardiovascular disease (37). Several mRNAs involved in cell-ECM or cell-cell interactions, with possible roles in mechanosensation, were differentially expressed between loaded and unloaded HISMCs. These included integrins, cadherins, catenins and catenin antagonists, claudins, a tight junction protein, talins, syndecans, an actinin, an adherens junction protein, cell adhesion molecules, and focal adhesion genes (Table 5). Finally, there were differential changes in mRNA encoding many cytoskeletal proteins (Table 6). These changes in gene expression suggest that, in response to cyclic stretching, HISMCs alter cell-cell and cell-ECM interactions, possibly consistent with a transition away from contractile SMC phenotypes.

### Loading induced expression of ligands that could signal to nearby cells.

**Figure 6:**
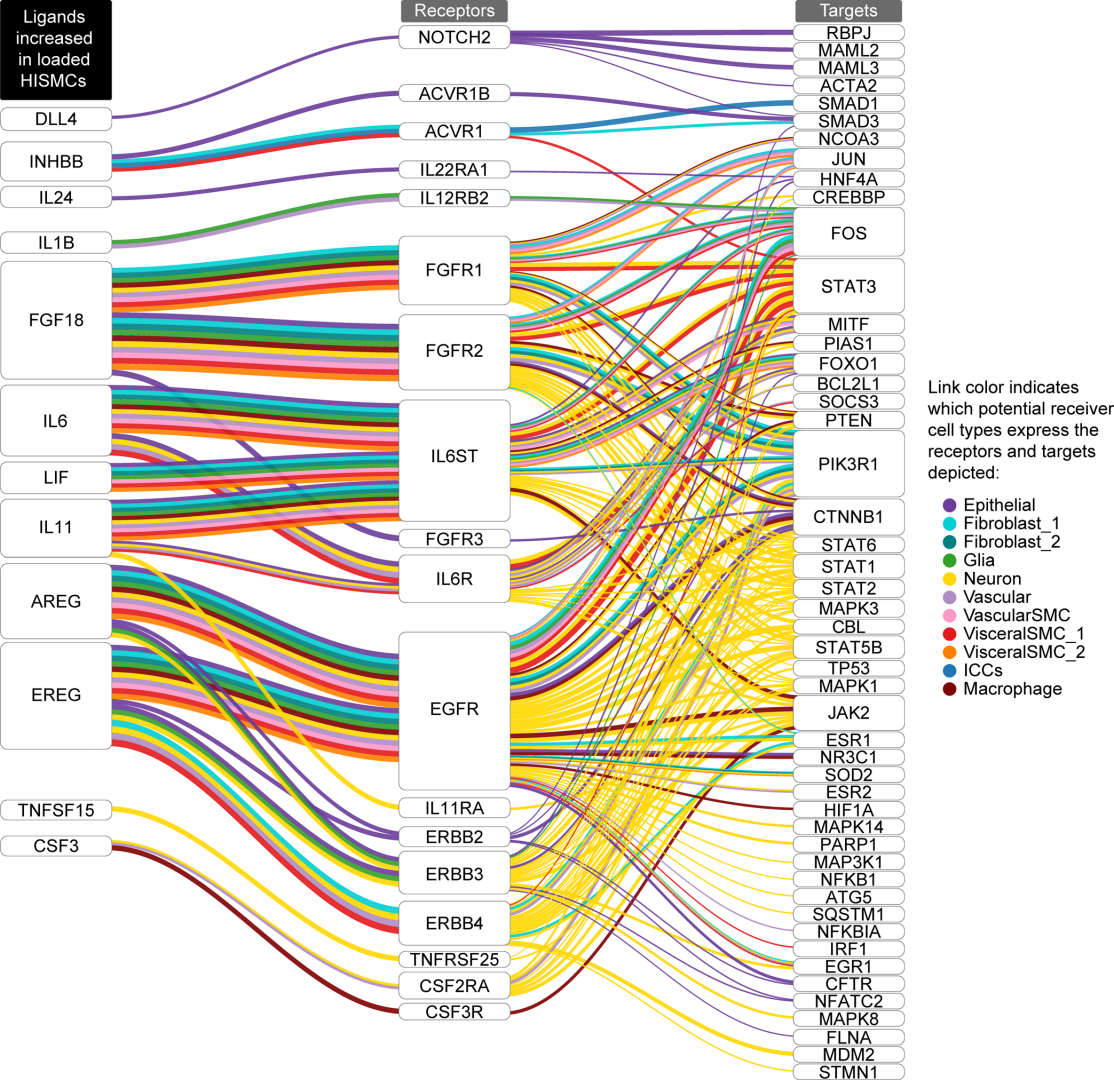
NicheNet ligand-receptor analysis using ligands more abundant in loaded HISMCs. Sankey plot showing potential ligand-receptor-target links based on NicheNet’s inferred signaling paths from “top 10” ligands upregulated in loaded HISMC Drokhlyansky et al. (39) receiver cell targets. NicheNet prioritized ligand analysis between secreted ligands more abundant in loaded compared with unloaded HISMCs (left column) and receptors (middle column) and target genes (right column) in reannotated Drokhlyansky et al. receiver cell types was used to infer signaling paths from each ligand to target. Potential ligand-receptor-target links were determined based on inferred signaling paths from NicheNet. See Methods and Supplemental Figure 3 for additional details on NicheNet analysis and process for inferring ligand-receptor-targets paths.

**Figure 7:**
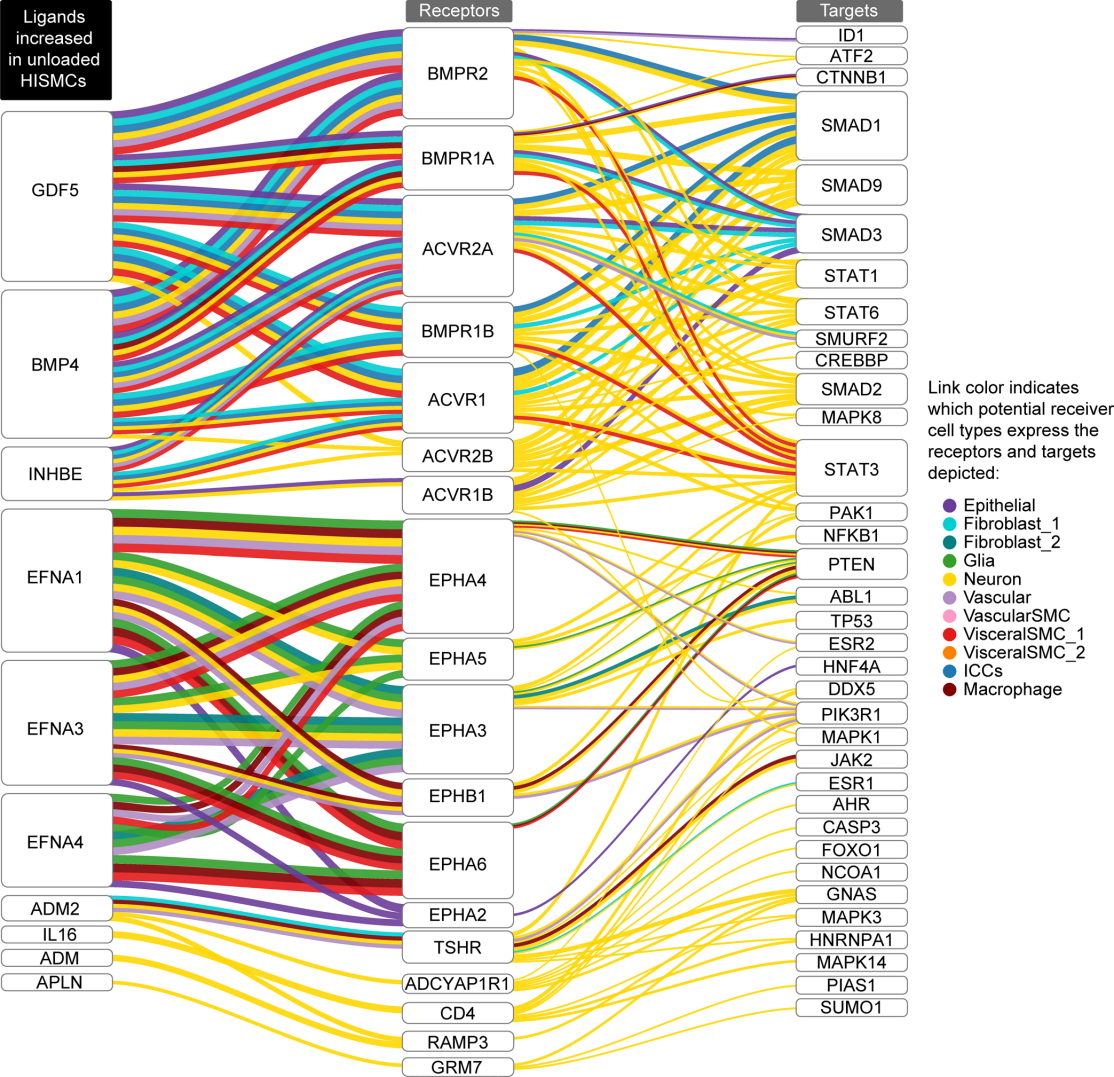
NicheNet ligand-receptor analysis using ligands more abundant in unloaded HISMCs. Sankey plot showing potential ligand-receptor-target links based on NicheNet’s inferred signaling paths from “top 10” ligands upregulated in unloaded HISMC to Drokhlyansky et al. (39) receiver cell targets. NicheNet prioritized ligand analysis between secreted ligands more abundant in unloaded compared with loaded HISMCs (left column) and receptors (middle column) and target genes (right column) in reannotated Drokhlyansky et al. receiver cell types was used to infer signaling paths from each ligand to target. Potential ligand-receptor-target links were determined based on inferred signaling paths from NicheNet. See Methods and Supplemental Figure 3 for additional details on NicheNet analysis and process for inferring ligand-receptor-targets paths.

Remarkably, many genes differentially regulated in HISMCs by cyclic loading encode secreted or cell surface ligands that could affect biology of nearby cells by binding cell surface receptors. To identify possible cellular targets for differentially expressed HISMC ligands, we used NicheNet (38) and human bowel single nucleus RNA-Seq data from Drokhlyansky et al. (39). The analysis strategy is summarized in Supplemental Figure 3. NicheNet evaluates potential ligand-receptor interactions, ranking interactions based on ligand-target regulatory potential (incorporating intracellular signaling into regulatory potential scoring). These potential ligand-receptor interactions are represented in Sankey plots (Figures 6 and 7). On the left of each Sankey plot is a ligand whose mRNA is more abundant in loaded HISMCs than in unloaded HISMCs (Figure 6), or conversely, a ligand more abundant in unloaded HISMCs than in loaded HISMCs (Figure 7), based on our data. In the middle column are receptors (from Drokhlyansky et al. data) (39) for ligands differentially expressed in our dataset. On the right of each Sankey plot are genes whose activity or expression is regulated by receptor signaling, according to the NicheNet model. The color of each line indicates the receptor-bearing cell type, based on Drokhlyansky et al. data (39). Figures 6 and 7 show the top 10 prioritized ligands from HISMCs (based on log_2_FC) for each cell type in a reannotated subset of Drokhlyansky et al. data. Additional data in Supplemental Figure 4 shows genes more abundant in loaded HISMCs for the next 10 prioritized ligands (Figure 6, Figure 7, and Supplemental Figure 4 show more than 10 ligands because top prioritized ligands differ between cell types). For example, *BMP4* mRNA is increased in unloaded compared with loaded HISMCs (as we confirmed in Figure 5C). The Sankey plot (Figure 7) shows that receptors for *BMP4* (i.e., *BMPR1A*, *BMPR1B*, and *BMPR2*) are expressed in visceral smooth muscle (VisceralSMC_1). However, *BMPR1A* is also expressed in enteric neurons, macrophage, Fibroblast_1, and epithelial cells, while *BMPR1B* is expressed in neurons, Interstitial cells of Cajal (ICC), and Fibroblast_1. The coreceptor *BMPR2* is expressed in VisceralSMC_1, vascular endothelial cells, enteric neurons, ICC, Fibroblast_1, and epithelial cells, but was not detected in macrophages in Drokhlyansky’s dataset (39). While some differentially expressed HISMC ligands could signal to many adjacent cell types (e.g., *FGF18*, *IL6*, *IL11*, *AREG*, *EREG*, *BMP2*, *HBEGF* in loaded HISMCs; *GDF5*, *BMP4*, *EFNA1*, *EFNA3*, *EFNA4* in unloaded HISMCs), other differentially expressed HISMC ligands were predicted to signal to only to neurons (*TNFSF15*, *IL16*, *INHBB* in loaded HISMCs; *ADM*, *APLN* in unloaded HISMCs) or to neurons, vascular endothelial cells, and macrophages (*CSF3* in loaded HISMCs). Notably, differentially expressed HISMC ligands from loaded cells have the largest number of targets in neurons, leading to the intriguing hypothesis that neurons may play an active role in how bowel responds to pathologic mechanical stress. For differentially expressed HISMC ligands in unloaded cells, there were fewer targets identified across all examined bowel cell types compared with the number of targets identified across bowel cell types for differentially expressed HISMC ligands in the loaded cells. Nevertheless, neuronal targets again feature prominently. Note that some possible interactions indicated in Sankey plots may not be biologically relevant (e.g., smooth muscle ICAM might never contact bowel epithelial cells). Nonetheless, these NicheNet analyses suggest that altered mechanical stress induces broad changes in HISMC gene expression and that many differentially expressed genes are likely to bind to receptors, and influence function, of other bowel wall cell types.

## Discussion

Mechanotransduction describes the ability of cells to actively sense, integrate, and convert mechanical stimuli into biochemical signals, including changes in transcription (40). Mechanotransduction is critical for normal bowel physiology and also affects disease pathophysiology in the context of pathologic mechanical stressors. Such pathologic force occurs in functional bowel obstruction (visceral myopathy, CIPO, Hirschsprung disease, ileus), during mechanical bowel obstruction (volvulus, adhesions, malignancy), and in the context of transmural inflammatory infiltrates or fibrosis (Inflammatory bowel disease, scleroderma). Mechanical stress sensing also affects symptoms in irritable bowel syndrome, functional dyspepsia, bowel diverticula, functional nausea, centrally mediated abdominal pain syndrome, dyssynergic defecation, achalasia, functional dysphagia, and visceral hypersensitivity (4). Although most bowel cell types appear capable of mechanotransduction (4), the effect of mechanical force on visceral smooth muscle phenotype remains underexplored.

Here, we tested the hypothesis that bowel smooth muscle phenotype might change in response to abnormal mechanical stress. Using cultured HISMCs, we show that even a short duration (6 hours) of pathological stretching (3% uniaxial cyclic stretch at 1 Hz) substantially altered expression of 4,537 HISMC genes, of which 2,500 met our minimum fold change threshold. The gene expression changes suggest mechanical loading induces HISMCs to transition to a synthetic, proinflammatory state. Predictive modeling with NicheNet further suggests that many genes induced by pathologic mechanical stress could act on a wide array of nearby cell types, causing complex long-lasting changes in bowel physiology. These gene expression changes induced in HISMCs by low-amplitude, high-frequency cyclical stretch are consistent with “phenotypic class switching,” a well-described phenomena in vascular smooth muscle (in which there are ≥ 9 known smooth muscle phenotypes) (41, 42). Phenotypic class switching is also reported in visceral smooth muscle, but studies are limited (6).

### Limitations.

All experiments employed a single HISMC line derived from human small intestine. Neither floating unloaded scaffolds nor cyclic stretching of loaded scaffolds truly mimic complex physical forces likely to be experienced by bowel smooth muscle in vivo. In vivo, bowel muscle experiences force from contraction and relaxation of muscle layers, pressure from intraluminal contents as adjacent bowel contracts or relaxes, force from movement of adjacent loops of bowel, movement with every breath as the diaphragm travels up and down, force from flow of blood through arterioles, and force every time an individual changes position. For example, a recent study showed loss of PIEZO1 mechanoreceptor in cholinergic neurons abolished exercise-induced acceleration of gut motility. This suggests mechanical force, when mice are running, normally activates PIEZO1, at least in neurons, and that this force alters bowel motility (43). Furthermore, during partial obstruction, surgical manipulation, pseudo-obstruction, stool impaction (e.g., constipation), Valsalva, enteric infection, inflammation (which slows bowel motility), or trauma, bowel smooth muscle experiences varied new mechanical stressors. While our studies do not recapitulate these mechanical stressors, they do demonstrate remarkable plasticity of HISMCs in response to even small amounts of mechanical force over short intervals.

Consistent with our data, unlike other muscle types, smooth muscle is not terminally differentiated. SMC phenotypic class switching is an unusual attribute, describing the ability of SMCs to reversibly modulate cell fate in response to various mechanical, chemical, and cytoskeletal triggers (7, 44). There are 2 well-recognized phenotypes for smooth muscle (contractile and synthetic) (6, 7, 41, 45), but in vascular SMCs, there are at least 9 identified SMC cell fates (42). These changes in SMC fate may be protective (e.g., forming a fibrous cap in damaged vasculature), but they are also implicated in pathophysiology in GI, cardiovascular, and pulmonary diseases (6, 33, 41, 46, 47). In some cases, prevention or reversal of SMC phenotypic class switching provides a therapeutic target (e.g., in atherosclerosis) (48–50). While underexplored, SMC phenotypic class switching may be an important mechanism and potential therapeutic target in some types of bowel disease (6). Understanding mechanisms that underlie visceral SMC phenotypic class switching may, therefore, provide avenues to prevent progression or reverse damage in human bowel disease.

### Mechanical stress induces phenotypic class switching from contractile to synthetic, proinflammatory HISMCs.

Contractile SMCs generate force needed for normal bowel motility. These SMCs may experience pathologic mechanical stress in many settings and appear to adapt over extended periods. For example, after surgical manipulation, bowel stops moving, a problem called “ileus” that typically lasts for several days. In contrast, mechanical obstruction leads to early occurrence of high frequency clustered contractions (3–10 regular contractions, occurring 1 contraction per 5 seconds, lasting < 1 minute, repeating every 1–3 minutes) called “minute rhythm” and “prolonged simultaneous contractions” (>8 seconds duration) (51, 52). These patterns also occur in intestinal neuropathy (1). The initial increase in motor activity after bowel obstruction is followed by suppression of motor activity and then by bowel muscle layer hypertrophy (1, 53). These observations reflect complex interactions between cell types and provide context for our HISMC data.

One striking observation is that loaded HISMC had 30% less *MKL2* and 76% less *CARMN* mRNA after only 6 hours of cyclic stretching (Figure 3I and Supplemental Table 1). MKL2 and *CARMN* are crucial for expression of contractile apparatus genes and are abundant in contractile phenotype SMCs (29, 54). At the same time, loaded HISMCs had more mRNA encoding proteins that block contractile apparatus gene expression or that induce synthetic/proliferative SMC phenotypes — e.g., *AREG* (increased 49.9-fold), *EREG* (increased 46.2-fold), and *KLF4* (increased 4.3-fold) (Table 1 and Supplemental Table 1), identified in vascular SMC literature (55–57). Loaded HISMCs had much more mRNA for proinflammatory cytokines, including *IL8* (increased 46.9-fold), *CXCL3* (increased 44.9-fold), *IL11* (increased 43.1-fold), *IL1B* (increased 40.8-fold), *PTGS2* (increased 32.0-fold), *IL6* (increased 13.4-fold), *ICAM1* (increased 3.6-fold), and *CCL2* (increased 2.1-fold), among other genes (Table 2 and Supplemental Table 1). Some of these observations fit with known signaling pathways. For example, IL-11 is produced by vascular smooth muscle in response to TGF-β1 and can act cell autonomously to induce phenotypic switching from contractile to synthetic, proinflammatory SMCs. In addition, IL-11–treated vascular SMCs increased gene expression for ECM and for *IL6* and *CCL2* (among other inflammatory mediators) (58). Interestingly, IL-11 protein abundance was not elevated in loaded HISMCs based on IHC. This discrepancy between mRNA and protein levels might occur because IL-11 is efficiently secreted, or might reflect regulation of translation by miRNA, altered IL-11 protein degradation, or increased time needed to translate mRNA to protein.

Similarly, IL-1β activates IL-1 receptors (expressed in HISMCs) (59), triggering nuclear localization of NF-κB (Figure 3, B and C). Nuclear NF-κB characteristically induces transcription of *ICAM1*, *CCL2* (also called *MCP1*), and *IL6*. This suggests cell-autonomous effects of IL-1β, produced in response to pathologic mechanical stress, could trigger many loading-induced changes in HISMC gene expression (60). NF-κB also mediates SMC phenotypic switching to a synthetic state (7, 19) by sequestering myocardin and preventing SRF-dependent expression of SMC contractile genes (61, 62). Consistent with our data, prior studies show static stretch (18%) increases SMC expression of *iNOS*, *IL6*, and *MCP1* within 3 hours, and bowel proximal to obstruction has markedly elevated PTGS2 (COX-2) after 24–48 hours (3, 10, 34). In addition, colon manipulation in vivo increases IL-1β within 24 hours (10). Collectively, these studies strongly support the hypothesis that “mechano-transcription” powerfully modulates gene expression in bowel smooth muscle and highlights complex, self-reinforcing networks that induce SMC phenotypic switching (1). These observations may have clinical implications for ileus, as well as for mechanical and functional bowel obstruction.

### Mechanical stress alters expression of many TGF-β family members in HISMC.

Many differentially expressed genes in loaded HISMCs encode TGF-β superfamily proteins or signaling pathways components (including *BMP2*, *BMP4*, *BMP6*, *GREM1*, *Noggin*, *BAMBI*, *INHBA*, *TGFB1*, *TGFBR1*, *ALK2* [reported as *ACVR1* in Supplemental Table 1]*,* and *ALK4* [reported as *ACVR1B* in Supplemental Table 1]). *TGFB1* mRNA increased 1.58-fold in loaded HISMCs, while receptors *TGFBR1* and *TGFBR3* mRNA increased ~1.9-fold in response to loading. Elevated TGF-β1 signaling also may explain the increase in IL-11 noted above. However, our analyses showed equivalent SMAD2/3 abundance in the nucleus of loaded and unloaded HISMCs, indicating no increase in TGF receptor signaling at this early (6 hour) time point.

We were also intrigued by the changes in BMP family mRNA because bowel smooth muscle patterning depends on the interplay of BMP2, BMP4, and BMP7, as elegantly shown by Huycke et al. (63). Furthermore, BMP2 increases vascular SMC migration and expression of synthetic markers (64, 65), and it has antiproliferative effects in pulmonary artery SMC (66). In addition, BMP2 counteracts many effects of TGF-β1 in SMCs by inducing PPARγ (67). While these observations are intriguing, our gene expression data (Table 3 and Supplemental Table 1) show increased *BMP2* and reduced *BAMBI* (BMP antagonist) mRNA in loaded HISMCs. Based on prior data, these changes should increase BMP receptor signaling. However, we also found reduced *BMP4*, and elevated *GREM1* and *NOG* (BMP antagonists) in loaded HISMCs, which should reduce BMP receptor signaling. To make sense of these observations, we looked for evidence of BMP signaling in HISMCs and found equivalent nuclear localization of SMAD1/5/8 in loaded and unloaded cells (Figure 5E), indicating no change in BMP receptor signaling. Collectively, these data suggest TGF-β superfamily signaling changes rapidly in response to mechanical stress in HISMCs, but these signaling systems were not (at least at this time point) affecting SMC cell phenotype; these data may also suggest that there is additional complexity not captured by our simplified system.

### Stress alters expression of axon guidance molecules and genes needed for cell-cell and cell-ECM interactions.

Many differentially expressed genes in loaded HISMCs encode axon guidance molecules (ephrins, netrins, semaphorins, and slits) that could influence bowel muscle innervation. Some encoded proteins also directly affect SMC biology, at least in vasculature. However, whether they are specifically related to contractile or synthetic phenotypes is not well understood. For example, ephrin B2 (*EFNB2*, increased 2.22-fold in loaded HISMCs) enhances vascular SMC contraction strength (68), while ephrin A1 (*EFNA1*, reduced 2.77-fold in loaded HISMCs) reduces integrin-induced vascular SMC spreading and inhibits SMC proliferation (69, 70). As another example, *SEMA7A* (increased 2.31-fold in loaded HISMCs) expression in vascular SMC is increased by PDGF (increased 2.04-fold in loaded HISMCs) and appears to be required for PDGF-induced vascular SMC proliferation and migration (71). In addition to these guidance molecules, which are not well studied in visceral SMCs, loading changed expression of many cytoskeletal proteins (or regulators like *FMN1*), integrins, cadherins, and focal adhesions, potentially altering SMC interactions with nearby cells and with ECM (Table 5).

### Pathologic stress–induced changes in HISMC gene expression could broadly affect the biology of many bowel cell types.

Although our studies employed purified HISMCs in culture, visceral smooth muscle cells in vivo closely interact with many other cell types including enteric neurons, glia, muscularis macrophages, fibroblasts, and vascular SMCs. HISMCs also interact very closely with ICC and PDGFRα^+^ cells to form the “SIP syncytium,” a network connected to SMCs by gap junctions (72–74). Recognizing many differentially expressed genes induced by loading in HISMCs encode secreted or extracellular ligands, we employed NicheNet to unravel potential SMC-niche interactions that might occur in response to pathologic stretching. Resulting analyses (Figures 6 and 7) suggest mechanotranscription responses to pathologic mechanical stress in HISMCs induce production of many growth factors (*EREG*, *AREG*, *HBEGF*, *FGF5*, *FGF7*, *FGF18*, *NRG1*, *PDGF*, *LIF*), cytokines (*IL6*, *IL11*, *CSF3*, *CLCF1*), and differentiation regulators (*WNT5A*, *BMP2*, *BMP6*, *DKK1*, *TGFB1*, *JAG1*, *DLL4*, *INHBB*, *INHBA*) likely to act on adjacent cells. For simplicity, our presented analyses include only the “Top 20” differentially expressed ligands in loaded versus unloaded HISMCs, based on the NicheNet model. Thus, these analyses show only a subset of the mechanotransduction-induced changes in HISMC gene expression. The interactions emphasize how physical stress experienced by HISMCs in a variety of disease or physiologic contexts could remodel not only smooth muscle but influence other bowel cell types. These other bowel cell types may also respond to the mechanical forces, thereby affecting gene expression patterns and interactions with each other and with visceral SMCs. Our reductive isolated HISMC culture system does not capture this complexity. These complex interactions may critically underlie some aspects of bowel dysfunction, especially for people with dysmotility or partial obstruction, where the broad array of cellular changes predicted to occur in response to mechanical stress might explain why recovery after bowel injury may be gradual (over days or months). Developing in vitro systems that better recapitulate both mechanics of physiologic human bowel contraction and capture interactions of various bowel cell types remains an active area of research for smooth muscle biology. One additional caveat is that our HISMCs were small bowel-derived while NicheNet analyses employed “receiver cell” mRNA abundance data from colon to use the most comprehensive single cell RNA-Seq dataset that included all cell types of interest. There may be region-specific effects of mechanical force in diverse bowel regions, a topic for future investigation.

### Conclusions.

We presented what we believe to be some of the first and most detailed analyses of gene expression data in HISMCs showing pathological mechanical stress, even over short time scales, leads to a switch toward a synthetic, proinflammatory HISMC phenotype. Future studies exploring the effect of these gene expression changes on protein abundance, intestinal smooth muscle function, intracellular signaling, and intercell communication may aid with clinical translation. Nonetheless, our NicheNet analyses generated testable hypotheses regarding the interplay between visceral smooth muscle and other bowel cell types that may occur in response to pathologic mechanical stress. These interactions may govern how bowel function is altered over long periods of time in human bowel diseases when such stresses are a substantial part of disease pathophysiology.

## Methods

### Sex as a biological variable.

Our study used human HISMCs that express *XIST* (gene count 1536 to 5403, based on our RNA-Seq data), indicating they were derived from a human female.

### Preparation of nanofibrous scaffolds.

Aligned and nonaligned poly(ε-caprolactone) (PCL) nanofibrous scaffolds (Mol. Wt. 80 kDa, Shenzhen Bright China Industrial Co. Ltd.) were fabricated via electrospinning, as described (75). Scaffolds were hydrated and sterilized in ethanol diluted in distilled water (100%, 70%, 50%, 30%; 30 min/step), and then incubated in laminin (20 μg/mL) (15, 76) solution in 1× Phosphate Buffered Saline (PBS) (Invitrogen, catalog 14190136) overnight (37°C) to enhance cell attachment.

### HISMC preparation and expansion.

In total, 5 × 10^5^ SMCs from human small intestine (cryopreserved at passage 1, ScienCell Research Laboratories, catalog 2910) were plated on 10 cm tissue culture dishes coated with 0.1% gelatin (MilliporeSigma, catalog G1890) and cultured (37°C, humidified incubator, 5% CO_2_) in HISMC media — SMC medium (ScienCell, catalog 1101), 2% FBS (fetal bovine serum, ScienCell, catalog 0010), 1% Penicillin/Streptomycin (ScienCell, catalog 0503), 1% SMC growth supplement (ScienCell, catalog 1152). HISMC media were changed every other day. Cells were passaged at 90% confluence. After 1–2 passages, confluent HISMCs were cryopreserved in 90% FBS/10% dimethyl sulfoxide (DMSO) at 5 × 10^5^ cells/mL. All experiments used HISMCs at passage 3–5.

### Dynamic mechanical loading of HISMC-seeded scaffolds.

Frozen HISMCs were thawed (37°C water bath, 2–3 minutes) and added to 10 mL Iscove’s Modification of DMEM (Corning, catalog 10-016-CM), and pelleted (270*g*, 3 minutes). Aligned laminin-coated PCL scaffolds (30 mm × 5 mm) were seeded with 350,000 HISMCs resuspended in 80 μL HISMC culture media. Cell-seeded scaffolds were maintained free-floating in HISMC media for 72 hours. “Loaded” scaffolds then experienced cyclic stretch (3% uniaxial stretch, 1 Hz, parallel to the long axis of HISMCs) for 6 hours in fresh HISMC media using a custom bioreactor (77). In parallel, “unloaded” HISMCs were maintained free-floating on scaffolds in fresh HISMC media for 6 hours. All cells were maintained at 37°C, 5% CO_2_ in a humidified incubator. Scaffolds were then cut in half. One half was dissolved in Trizol (Ambion, catalog 15596018) for RNA extraction and the other half fixed for IHC.

### RNA extraction and purification.

To isolate RNA from HISMCs on PCL scaffolds, scaffolds were minced in 500 μL TRizol (Ambion, catalog 15596018) using sharp scissors and vortexed up to 15 minutes until scaffold dissolved. RNA was purified from cells lysed in TRIzol using RNeasy Plus Mini kit (QIAGEN, catalog 74134), with RNase Free DNase Set (QIAGEN, catalog 79254) to remove residual DNA. RNA concentrations were measured by NanoDrop (ND-2000, Thermo Fisher Scientific).

### qPCR.

qPCR used SsoFast Evagreen Supermix with Low ROX (Bio-Rad, catalog 172-684 5211). Primers are in Supplemental Table 2. Cycle threshold (C_t_) values were normalized to *YWHAZ* mRNA. Data are plotted as 2^ΔCt^ = 2^(Ct^ ^[mRNA^ *^of^* ^interes]^^–^^Ct^ ^[*YWHAZ*])^.

### Immunofluorescence staining.

Scaffolds were washed once with 1× PBS, fixed (4% paraformaldehyde, 30 minutes, room temperature), washed twice with 1× PBS (5 minutes each, room temperature), blocked (5% normal donkey serum [NDS], 0.5% Triton X-100 in PBS (0.5% PBST), 1 hour, room temperature), incubated in primary antibodies (5% NDS, 0.5% PBST 1 hour, room temperature) (Supplemental Table 2), washed 3 times for 5 minutes (0.5% PBST), and then incubated in secondary antibodies (Supplemental Table 3) (0.5% PBST, 30 minutes, dark, room temperature). Phalloidin staining was performed after secondary antibody staining by washing 3 times for 5 minutes (PBS) and incubating (1 hour, dark, room temperature) in Alexa Fluor–conjugated phalloidin (488 nm, 555 nm, or 647 nm; Invitrogen catalog A12379, A34055, and A2287) diluted 1:1,000 in PBS. Cells were washed 2 times in 1× PBS, incubated in 1:30,000 SYTOX green (Thermo Fisher Scientific, catalog S7020) diluted in Hanks Balanced Salt Solution (30 minutes, dark, room temperature), washed twice in 1× PBS, mounted in Prolong Diamond AntiFade Mountant (Thermo Fisher Scientific, Catalog#P36961), and allowed to set (overnight, dark, room temperature) before storage in PBS at 4°C.

### Immunofluorescence microscopy.

Scaffolds were imaged using a Zeiss LSM 710 (Zeiss ZEN 2.3 SP1 FP3, black; version 14.0.18.201; data in Figure 1) or LSM 980 (Zeiss Zen Blue 3.5 software; data in all other figures) laser scanning confocal microscopes. Images were acquired with a ×20/0.8 air or ×63/1.4 oil DIC M27 Plan-Apochromat objective. Confocal images were processed in ImageJ (NIH) to crop, scale, and uniformly color adjust. Confocal images are represented as “sum of slices” or “maximum intensity” projections after ImageJ processing.

### Quantitative image analysis.

MYH11 fluorescence intensity quantitative analysis employed Imaris software (version 9.02, Bitplane Inc.). Phalloidin staining was used to generate isosurfaces corresponding to cells. Cell volume and total MYH11 fluorescence intensity were obtained from isosurfaces and fluorescence intensity normalized to cell volume, as reported (78). IL-11 quantification was done in a similar manner, with normalization of fluorescence intensity normalized to cell volumes. For nuclear to cytoplasmic intensity ratio calculations, images were processed using ImageJ (NIH). Z stacks containing identified cells were condensed to “sum of slices” projections. Cells and nuclei were outlined using “freehand selection tool” and intensity measured for each antibody. Parameters calculated included raw intensity, volume of cell and nuclei.

### Bulk RNA-Seq analyses.

Libraries prepped using TruSeq total RNA-Seq kit (Illumina, 20020596; TruSeq Stranded Total RNA Library Prep Human/Mouse/Rat; 48 Samples) were sequenced (paired-end) on Illumina NovaSeq 6000. Bioinformatics pipeline nf-core/rnaseq (79) — reference genome: GRCh38, aligner: STAR (80), quantifier: RSEM (81) — provided counts for 29,972 genes. Additional analyses of bulk RNA-Seq gene count data were performed in R (v4.4) (82) using RStudio Server (2023.06.1 Build 524) (83). Gene count data were filtered using *WGCNA* (84) function goodSamplesGenes() with default parameters to remove genes with too many missing entries across samples, resulting in 20,410 remaining genes. Additional filtering removed genes with fewer than 50 counts across all samples. Remaining 14,892 genes were used for downstream analysis. *DESeq2* (v1.44, Wald test method, contrasting loaded versus unloaded samples, and using the log_2_ fold change [log_2_FC] shrinkage method; Benjamini-Hochberg procedure for FDR) was used for differential gene expression analysis to compare loaded versus unloaded conditions (85).

### GSEA.

GSEA employed *fgsea* (v3.17) (86) in R. Genes were ranked based on log_2_FC from differential expression analysis of loaded versus unloaded HISMCs. NES reflect the degree to which genes are overrepresented at the top or bottom of entire ranked gene lists, normalized to mean enrichment of random samples of the same size. The Human MSigDB Collections Hallmark (MSigDB v7.5.1) (87, 88) gene sets were used for this analysis (87–89). MSigDB human gene sets were downloaded via the R package *msigdbr* (v7.5.1) (88).

### STRING analyses.

STRING is a database of known and predicted protein-protein interactions. This includes direct/physical and indirect/functional associations (90). STRING (v11.5) was used to examine possible interactions between differentially expressed genes based on DESeq2 analyses. STRING input included 500 genes with lowest adjusted *P* values filtered for log_2_FC < –0.48 or > 0.48. Pathway enrichment analysis in STRING software used “whole genome” background option in STRING for statistical comparison employing KEGG and GO Pathways.

### Ligand-receptor association analyses using NicheNet.

NicheNet (*nichenetr*, v2.0.0) was employed to characterize potential interactions between HISMC-expressed ligands and receptors present in various bowel cell types (38). NicheNet prioritizes ligands in “sender/niche” populations most likely to affect (according to the NicheNet model) transcriptional states of gene sets of interest in “target/receiver” populations. For our analyses, “sender/niche” genes were genes differentially expressed (identified in DESeq2 analysis) in HISMCs cultured 6 hours on loaded versus unloaded scaffolds, filtered for adjusted *P* value less than 0.05 and log_2_FC < –0.48 (for “up in unloaded”; 14 ligands) or > 0.48 (for “up in loaded”; 57 ligands). Gene symbols were converted to official HUGO Gene Nomenclature Committee at the University of Cambridge (HGNC) symbols using GeneSymbolThesarus() function in *Seurat* (v4.4.0) (91) prior to NicheNet analysis. NicheNet was run separately for ligands “up in loaded” and ligands “up in unloaded.” Selected cell types from human single nucleus RNA-Seq (droplet-based MIRACL-seq) dataset published by Drokhlyansky et al. were used as “receiver/target” populations (39). Patient-specific clusters (“H3,” “MHC.I_H1,” “MHC.I_H9,” “OXPHOS_H3”) described by Drokhlyansky et al. were removed from dataset prior to analysis. The following cell types from Drokhlyansky et al. were used for this analysis with original cell type annotations: Epithelial, Fibroblast_1, Fibroblast_2, ICCs, Macrophage, and Neuron. Myocyte clusters from Drokhlyansky et al. were reannotated for our analysis based on differential gene expression reported by ref. 39. According to the average log_2_FC reported in Supplemental Table 4 of Drokhlyansky et al., Myocyte_3, Myocyte_4, and Myocyte_5 all had MYH11, suggesting these myocyte clusters are smooth muscle clusters. Myocyte_3 and Myocyte_5 had more ACTG2 than ACTA2, suggesting these are visceral SMC clusters (reannotated as VisceralSMC_1 and VisceralSMC_2, respectively), and Myocyte_4 had more ACTA2 than ACTG2, suggesting this is a vascular SMC cluster (reannotated as VascularSMC). The following cell type groups were generated and used for this analysis by grouping and renaming original cell type annotations: Glia (includes Glia_1, Glia_2, and Glia_3) and Vascular (includes Vascular_1 and Vascular_2, probably endothelial cells based on marker gene expression). Gene sets of interest for each “receiver/target” cell type were defined as differentially expressed genes identified using the FindMarkers function (Wilcoxon Rank Sum test) in Seurat (91). Genes were only considered for differential expression testing if expressed in at least 10% of cells in that population and for differential expression for each cell type compared each cell type against all other cell types in the Drokhlyansky et al. dataset (39). Resulting differentially expressed genes were filtered to keep only genes with adjusted *P* < 0.05 and average log_2_FC > 0.25 or < –0.25. These filtered results were used as receiver gene sets of interest in NicheNet analysis. The NicheNet prior model (v2) was modified according to developer “model construction” instructions to keep only data sources classified as “literature” and “comprehensive_db.” NicheNet ranks potential ligands based on the presence of receptors and target genes in gene sets of interest that are associated with each ligand in the NicheNet model (compared with background of genes for that cell type; background genes were identified for each cell type individually via the get_expressed_genes() function in NicheNet with default parameters). For each cell type, results of ligand activity analysis were filtered based on log_2_FC (from the DESeq2 differential expression analysis), keeping only the “top 20” ligands (or fewer if not more than 20) by absolute value of log_2_FC for each receiver cell type. To infer potential ligand-to-target signaling paths, the get_ligand_signaling_path() function in NicheNet was run for each set of ligands and receiver cell type. The inferred signaling network for each receiver cell type was filtered to remove target genes if they were also identified as receptors within a particular cell type and to keep only ligand-receptor, ligand-target, and receptor-target links that contributed to a complete ligand-receptor-target signaling path. Sankey plots of inferred ligand-receptor-target paths were generated using subsets of the “top 20” ligands results for each receiver cell type, separated into “top 10” (ligands 1–10, ranked by greatest fold change) and “next 10” (ligands 11–20, ranked by greatest fold change). As these subsets of ligands were generated individually for each receiver cell type, “top 10” and “next 10” refers to top ligands prioritized for each receiver cell type, and prioritized ligands are not identical for all receiver cell types. A schematic for how NicheNet was used for this analysis is in Supplemental Figure 3. Sankey plots were generated using R package *sankeyD3* (v0.3.2) (92) and manually edited in Adobe Illustrator (2023) for legibility.

### Statistics.

GraphPad Prism (version 9.5.1) was used for statistical analysis of qPCR and quantitative image analysis data. Two-tailed Student’s *t* test (parametric data) or Mann-Whitney *U* tests (nonparametric data) were used for comparisons between 2 groups. *P* < 0.05 was considered significant. Data are represented as mean ± SEM for parametric data and median (interquartile range) for nonparametric data. Statistical analysis of RNA-seq data was performed as discussed below.

### Study approval.

This study used commercially available cell lines and reagents. No study approvals were required.

### Data availability.

A Supporting Data Values file is available in supplemental materials. Full data sets are deposited in Gene Expression Omnibus (GEO) accession no. GSE264225. Differentially expressed gene lists can be accessed in Supplemental Table 1. All code and package version information used for DESeq2, GSEA, and ligand-receptor-target NicheNet analyses is available on GitHub at github.com/HeuckerothLab/Mechanobiology_Wolfson2024/ (version tag v1.0.0).

## Author contributions

Conceptualization was contributed by SMW, SCH, ROH, and SKH. Methodology was contributed by SMW, KB, ROH, and SKH. Investigation was contributed by SMW, KB, SEA, BD, SHL, MW, SCH, ROH, and SKH. Formal analysis was contributed by SMW, KB, BD, ROH, and SKH. Data curation was contributed by SMW, KB, BD, ROH, and SKH. Writing of original draft was contributed by SKH and SMW. Review and editing were contributed by SMW, KB, SEA, BD, MW, SHL, DMT, SCH, ROH, and SKH. Resources were contributed by SKH, SMW, DMT, SCH, and ROH. Supervision was contributed by SKH, DMT, SCH, and ROH. Funding acquisition was contributed by SCH, ROH, and SKH.

## Funding support

This work is the result of NIH funding, in whole or in part, and is subject to the NIH Public Access Policy. Through acceptance of this federal funding, the NIH has been given a right to make the work publicly available in PubMed Central.

- NIH F30DK118827 (SKH)
- NIH R01DK129691 (ROH)
- Irma and Norman Braman Endowment (ROH)
- Suzi and Scott Lustgarten Center Endowment (ROH)
- The Children’s Hospital of Philadelphia Frontier Program for Precision Diagnosis and Therapy for Pediatric Motility Disorders (ROH)
- The Children’s Hospital of Philadelphia Research Institute (ROH)
- The NSF Science and Technology Center for Engineering Mechanobiology (CMMI-1578571).

## Supplementary Material

Supplemental data

Supplemental table 1

Supporting data values

## Figures and Tables

**Table 1:**
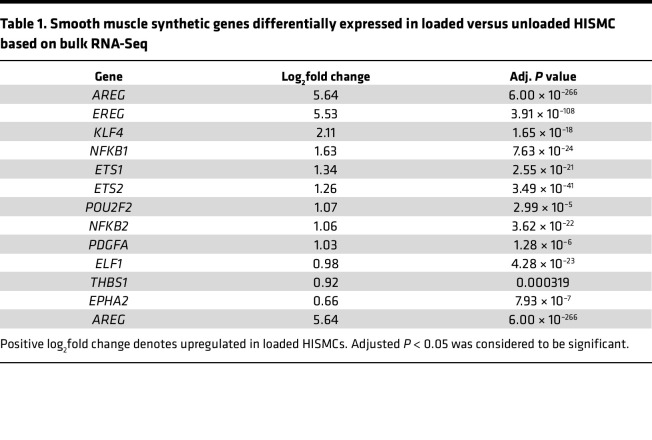
Smooth muscle synthetic genes differentially expressed in loaded versus unloaded HISMC based on bulk RNA-Seq

**Table 2:**
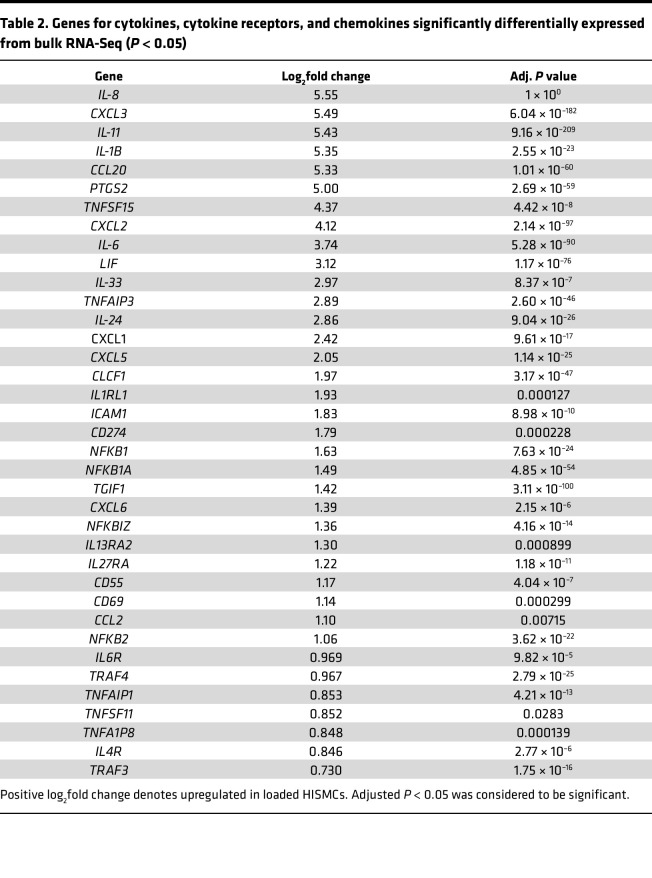
Genes for cytokines, cytokine receptors, and chemokines significantly differentially expressed from bulk RNA-Seq (*P* < 0.05)

**Table 3:**
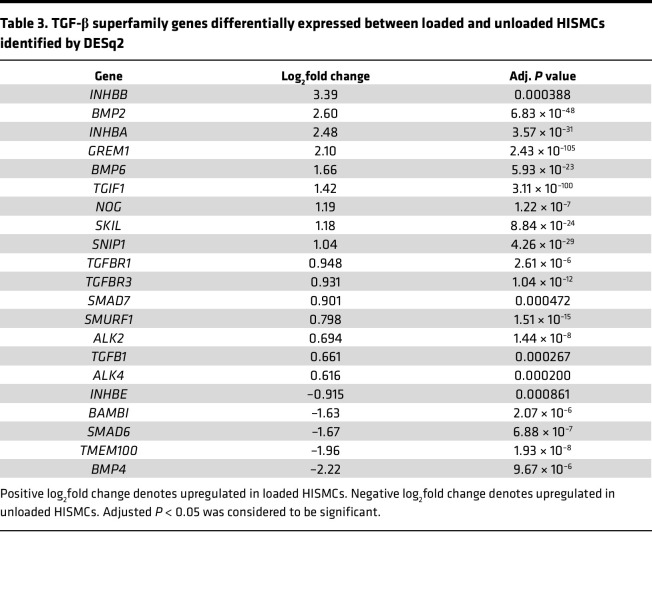
TGF-β superfamily genes differentially expressed between loaded and unloaded HISMCs identified by DESq2

**Table 4:**
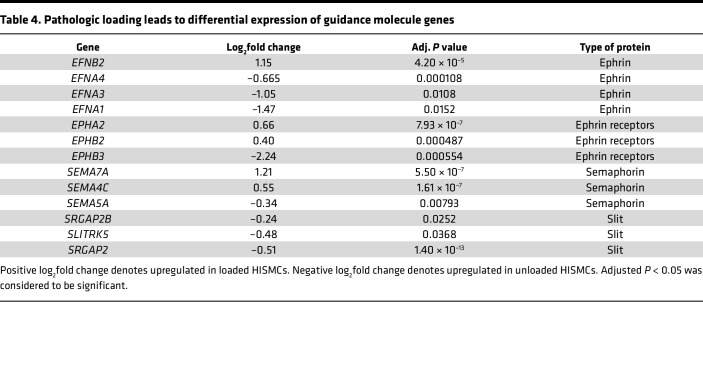
Pathologic loading leads to differential expression of guidance molecule genes

**Table 5:**
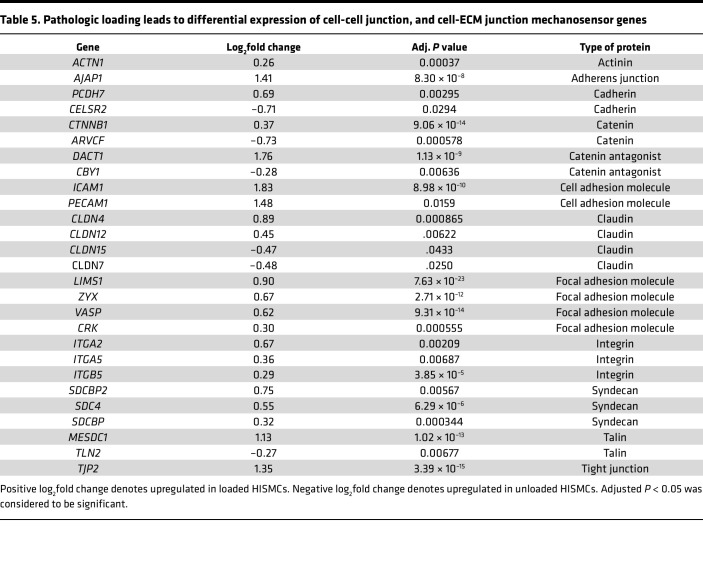
Pathologic loading leads to differential expression of cell-cell junction, and cell-ECM junction mechanosensor genes

**Table 6:**
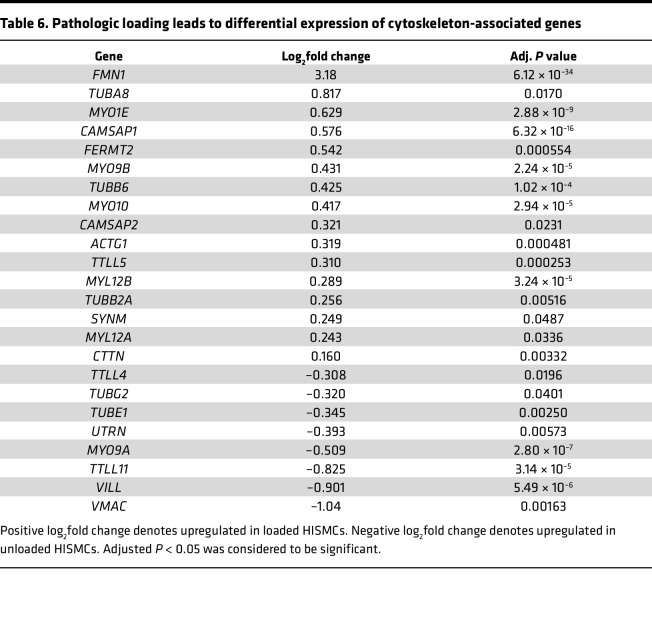
Pathologic loading leads to differential expression of cytoskeleton-associated genes

## Version 1 09/16/2025

In-Press Preview

## Version 2 10/22/2025

Electronic publication
