# Supplementary material for: Rapid cyclic stretching of cultured human visceral smooth muscle cells promotes a synthetic, proinflammatory phenotype
Source: JCI Insight. 2025 Sep 16;10(20):e188669. doi: 10.1172/jci.insight.188669 (PMC12581679; doi:10.1172/jci.insight.188669)
Supplement: Supplemental data [file jciinsight-10-188669-s258.pdf]

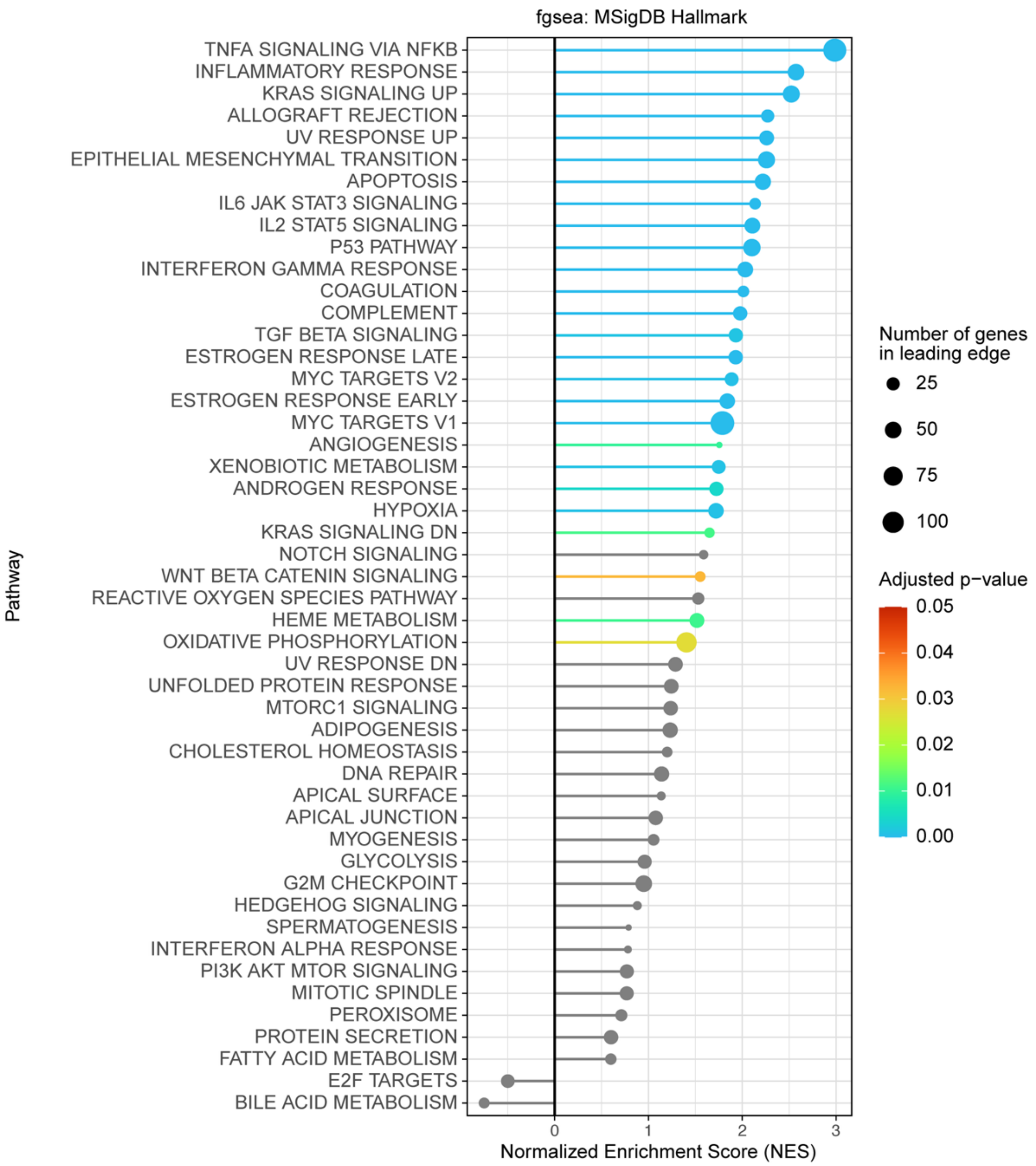

Supplemental Figure 1.
Gene set enrichment (fgsea) results for MSigDB Hallmark Pathways, using the differentially expressed genes between loaded and unloaded HISMCs.

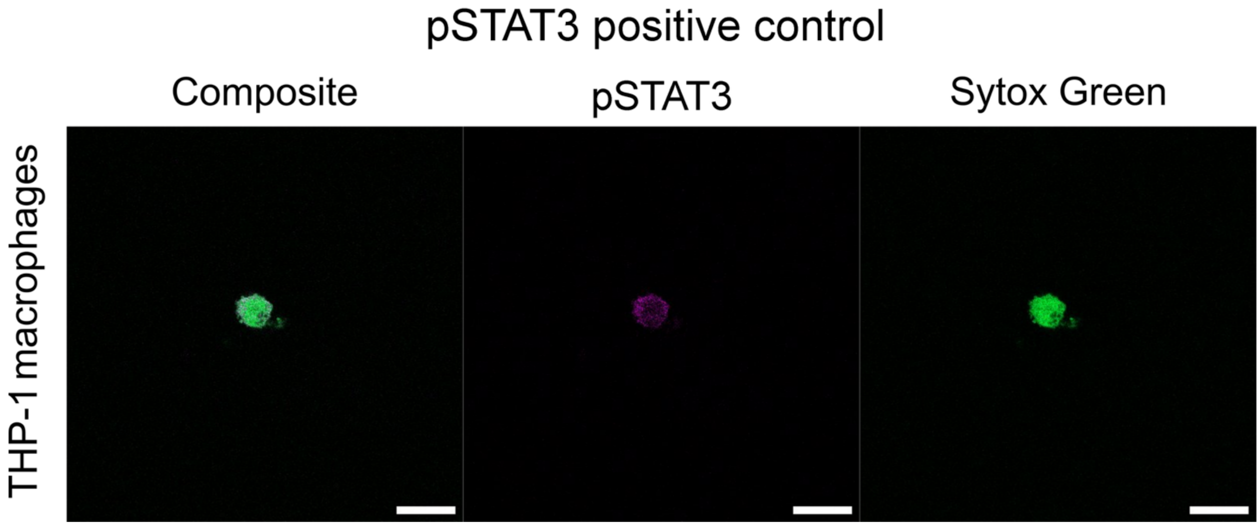

Supplemental Figure 2.
The presence of phospho-STAT3 (PSTAT3) staining was confirmed in THP-1 macrophages.

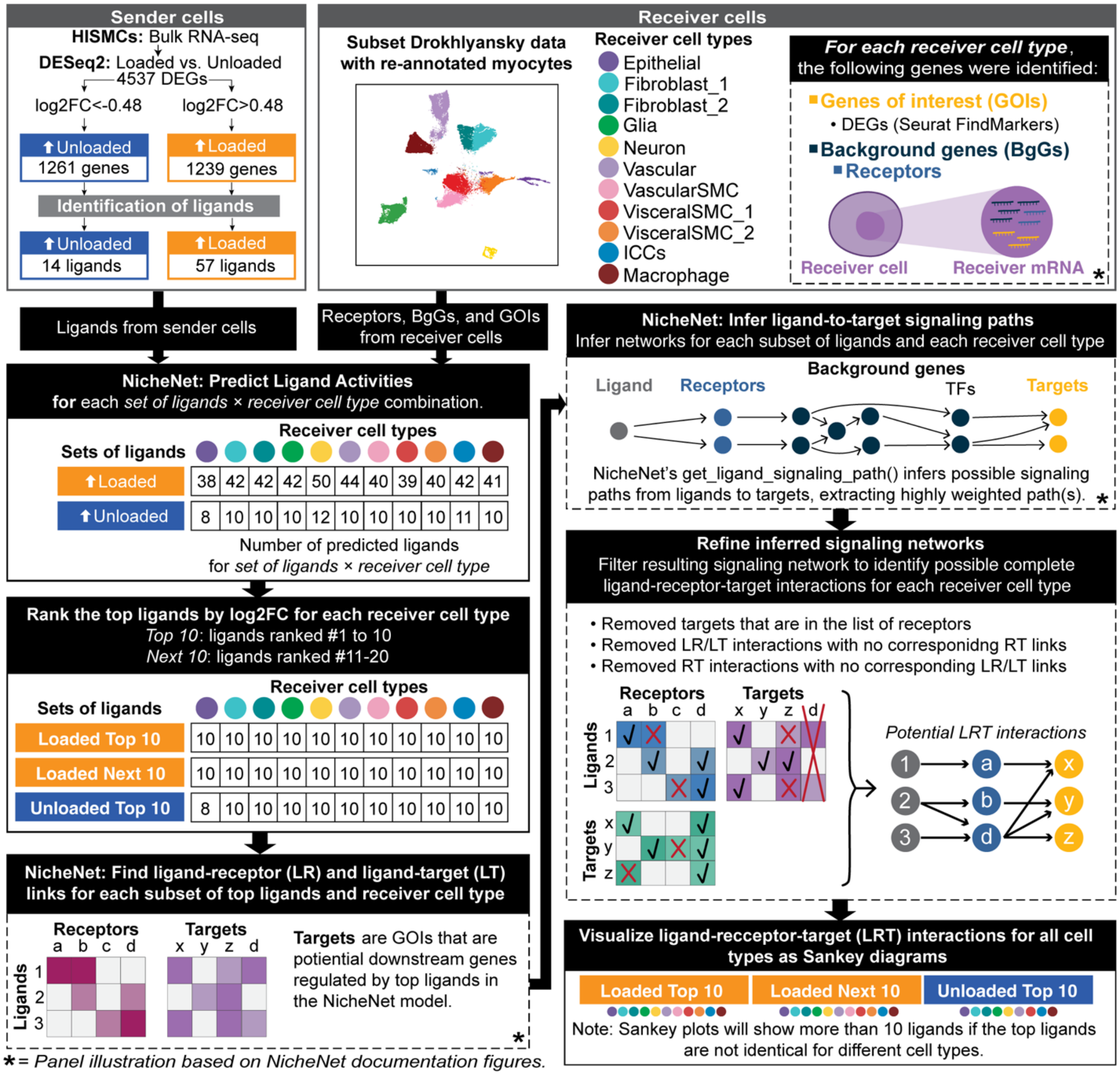

Supplemental Figure 3.
Schematic overview of the NicheNet analysis methods, including the process for filtering the inferred signaling network to keep only the potential interactions with ligand-receptor-target paths.

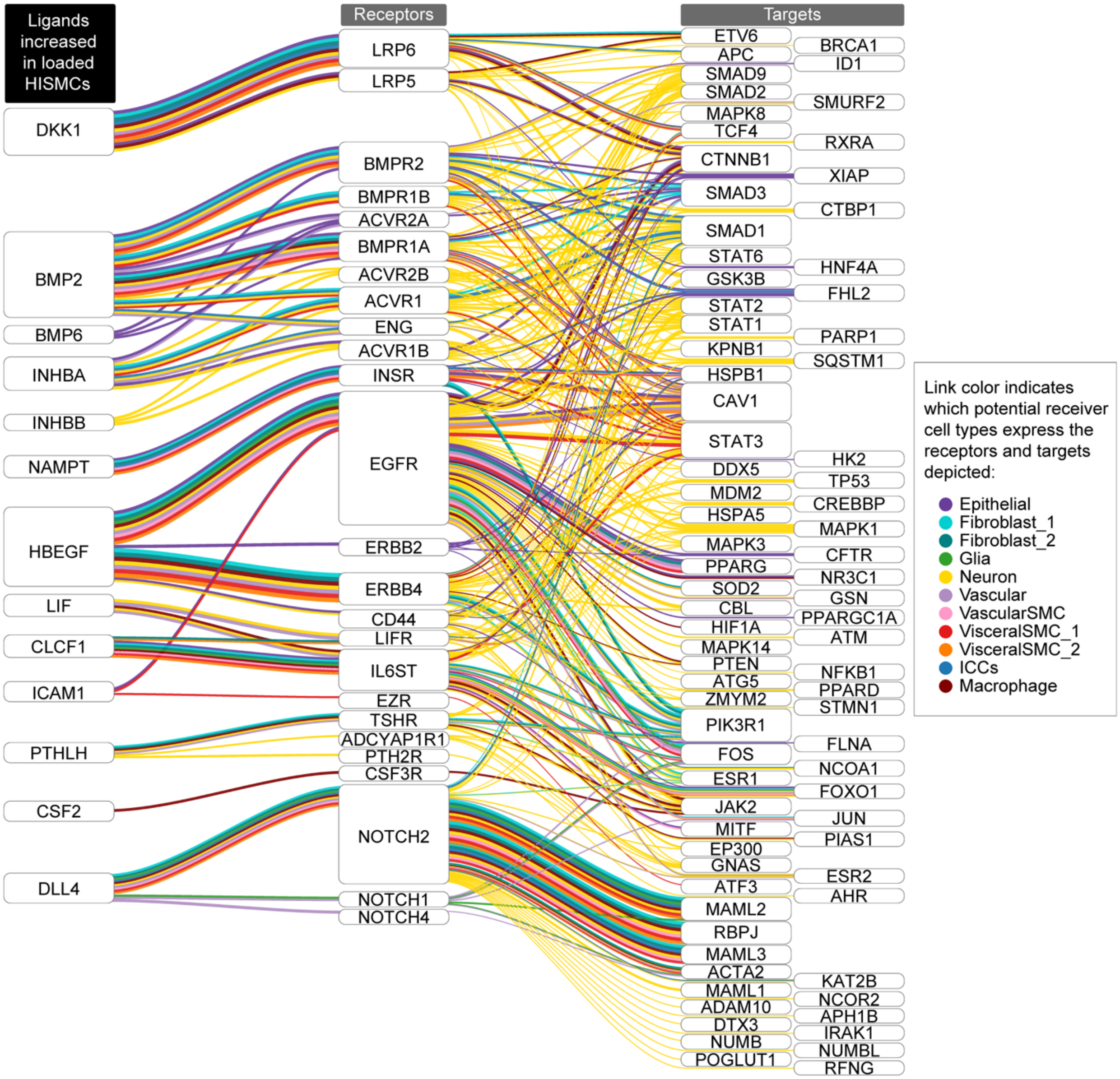

Supplemental Figure 4.

Sankey plot showing potential ligand-receptor-target links based on NicheNet's inferred signaling paths from the "next 10" ligands upregulated in the loaded HISMC to Drokhlyansky et al. receiver cell targets. The NicheNet prioritized ligand analysis between secreted ligands that are more abundant in loaded compared to unloaded HISMCs (left column) and receptors (middle column) and target genes (right column) in re-annotated Drokhlyansky et al. receiver cell types was used to infer signaling paths from each ligand to target. Potential ligand-receptor-target links were determined based on the inferred signaling paths from NicheNet. See Methods and Supplemental Figure 1 for additional details on the NicheNet analysis and process for inferring ligand-receptor-targets paths.

Supplemental Table 1.
This is a separate file with all DESeq2 differentially expressed genes.

Supplemental Table 2. Primers.

Primer Name	Sequence (5' to 3')	Tm (˚C)	GC (%)
YWHAZ Fwd	TGCTTGCATCCCACAGACTA	57	50
YWHAZ Rev	AGGCAGACAATGACAGACCA	57	50
ACTG2 Fwd	CATGTACGTCGCCATTCAAGC	58	52
ACTG2 Rev	TTGATGTCTCGCACAATTTCTCT	56	39
ACTA2 Fwd	CACTGTCAGGAATCCTGTGA	55	50
ACTA2 Rev	CAAAGCCGGCCTTACAGA	56	56
MYH11 Fwd	AGATGGTTCTGAGGAGGAAACG	58	50
MYH11 Rev	AAAACTGTAGAAAGTTGCTTATTCACT	55	33
IL6 Fwd	GGAGACTTGCCTGGTGAAA	62	53
IL6 Rev	CTGGCTTGTTCCTCACTACTC	62	50
IL11 Fwd	GAGAGGCTTGCTTGGGATATAG	62	50
IL11 Rev	TCCCAAAGTGCCAGGATTAC	62	50
GREM1 Fwd	GCAGGATAGTGGAGTGAGAAAG	62	50
GREM1 Rev	TCAGCCTGTGTTCTGGTATTG	62	48
BMP2 Fwd	GAGAAGGAGGAGGCAAAGAAA	62	48
BMP2 Rev	GGGACACGTCCATTGAAAGA	62	50
BMP4 Fwd	GGAGATGGTAGTAGAGGGATGT	62	50
BMP4 Rev	CGTGTGTGTGTGGTGTATGT	62	50
COL1A1 Fwd	GAGGGCCAAGACGAAGACATC	62	57
COL1A1 Rev	CAGATCACGTCATCGCACAAC	62	52
FN1 Fwd	CGGTGGCTGTCAGTCAAAG	61	58
FN1 Rev	AAACCTCGGCTTCCTCCATAA	61	48
VIM Fwd	GCCCTAGACGAACTGGGTC	61	63
VIM Rev	GGCTGCAACTGCCTAATGAG	61	55
MMP14 Fwd	GGCTACAGCAATATGGCTACC	60	52
MMP14 Rev	GATGGCCGCTGAGAGTGAC	62	63

Supplemental Table 3. Antibodies and staining reagents.

**Antibodies (species)**	**Application (Concentration)**	**Manufacturer**	**Catalog#, RRID**
Anti-phosphoSmad1/Smad5/Smad8, phospho-specific (Ser463/465) (rabbit)	Immunofluorescence (1:500)	Millipore	AB3848, RRID:AB_177439
Smad2 (pS465/pS467)/Smad3 (pS423/pS425) (mouse)	Immunofluorescence (1:500)	BD Phosflow	562586, RRID:AB_11151915
IL11 (rabbit)	Immunofluorescence (1:200)	Invitrogen	PA5-36544, RRID:AB_2553579
NF- B p65 (L8F6) (mouse)	Immunofluorescence (1:1000)	Cell Signaling	6956, RRID:AB_10828935
Smooth Muscle Myosin Heavy Chain (SMMS-1) (mouse)	Immunofluorescence (1:100)	Abcam	ab106919, RRID:AB_10866244
Phospho-Stat3 (Tyr705) (D3A7) (rabbit)	Immunofluorescence (1:200)	Cell Signaling	9145, RRID:AB_2491009
Megakaryoblastic Leukemia 2 (MLK2) (rabbit)	Immunofluorescence (1:200)	Novus Bio	NBP1-46209, NP_054767.3
**Secondary Antibodies**			
Donkey anti-mouse 594	Immunofluorescent (1:400)	Invitrogen	A21203, RRID AB_2535789
Donkey anti-rabbit 647	Immunofluorescent (1:400)	Invitrogen	A31573, RRID AB_2536183
Donkey anti-mouse 647	Immunofluorescent (1:400)	Invitrogen	A3157, RRID AB_162542
Donkey anti-rabbit 594	Immunofluorescent (1:400)	Invitrogen	A21207, RRID AB_141637
Donkey anti-chicken 594	Immunofluorescent (1:400)	Invitrogen	A11042, RRID AB_2534099
**Other staining reagents**			
Sytox Green nucleic acid stain	Immunofluorescence (1:30000)	ThermoFisher	S7020, RRID: not available
Alex Fluor 647 Phalloidin	Immunofluorescence (1:1000)	Invitrogen	A22287 RRID:AB_2620155
